# Machine Learning and Deep Learning Hybrid Approach Based on Muscle Imaging Features for Diagnosis of Esophageal Cancer

**DOI:** 10.3390/diagnostics15141730

**Published:** 2025-07-08

**Authors:** Yuan Hong, Hanlin Wang, Qi Zhang, Peng Zhang, Kang Cheng, Guodong Cao, Renquan Zhang, Bo Chen

**Affiliations:** 1 Department of General Surgery, The First Affiliated Hospital of Anhui Medical University, Hefei 230022, China; hongyuancsgo@163.com (Y.H.);; 2 Department of Thoracic Surgery, The First Affiliated Hospital of Anhui Medical University, Hefei 230022, China; whl17318594298@163.com (H.W.); 18712531026@163.com (Q.Z.); 3 Department of The First Clinical Medical College, Anhui Medical University, Hefei 230022, China

**Keywords:** esophageal cancer, radiomics, machine learning, deep learning, multimodal imaging

## Abstract

**Background**: The rapid advancement of radiomics and artificial intelligence (AI) technology has provided novel tools for the diagnosis of esophageal cancer. This study innovatively combines muscle imaging features with conventional esophageal imaging features to construct deep learning diagnostic models. **Methods**: This retrospective study included 1066 patients undergoing radical esophagectomy. Preoperative computed tomography (CT) images covering esophageal, stomach, and muscle (bilateral iliopsoas and erector spinae) regions were segmented automatically with manual adjustments. Diagnostic models were developed using deep learning (2D and 3D neural networks) and traditional machine learning (11 algorithms with PyRadiomics-derived features). Multimodal features underwent Principal Component Analysis (PCA) for dimension reduction and were fused for final analysis. **Results**: Comparative analysis of 1066 patients’ CT imaging revealed the muscle-based model outperformed the esophageal plus stomach model in predicting N2 staging (0.63 ± 0.11 vs. 0.52 ± 0.11, *p* = 0.03). Subsequently, multimodal fusion models were established for predicting pathological subtypes, T staging, and N staging. The logistic regression (LR) fusion model showed optimal performance in predicting pathological subtypes, achieving accuracy (ACC) of 0.919 in the training set and 0.884 in the validation set. For predicting T staging, the support vector machine (SVM) model demonstrated the highest accuracy, with training and validation accuracies of 0.909 and 0.907, respectively. The multilayer perceptron (MLP) fusion model achieved the best performance among all models tested for N staging prediction, although the accuracy remained moderate (ACC = 0.704 in the training set and 0.685 in the validation set), indicating potential for further optimization. Fusion models significantly outperformed single-modality models. **Conclusions**: Based on CT imaging data from 1066 patients, this study systematically constructed predictive models for pathological subtypes, T staging, and N staging of esophageal cancer. Comparative analysis of models using esophageal, esophageal plus stomach, and muscle modalities demonstrated that muscle imaging features contribute to diagnostic accuracy. Multimodal fusion models consistently showed superior performance.

## 1. Introduction

Esophageal cancer represents a significant global health challenge and ranks among the leading causes of cancer-related deaths worldwide. It primarily consists of two histological subtypes: esophageal squamous cell carcinoma, prevalent in East Asia and parts of Africa, and esophageal adenocarcinoma, whose incidence has been rising in Western countries [1,2]. Recent advancements in diagnostic techniques have significantly improved early detection rates and staging accuracy for esophageal cancer. Currently, innovations in minimally invasive surgery and optimized neoadjuvant chemoradiotherapy protocols have notably enhanced surgical outcomes and patient survival rates [3]. Nevertheless, due to its subtle or atypical early symptoms, esophageal cancer is often diagnosed at advanced stages when patients experience dysphagia or obstructive symptoms, making early diagnosis an ongoing clinical challenge [4].

In recent years, significant progress has been made in computed tomography (CT)-based radiomics. This technology involves extracting and analyzing high-dimensional quantitative features from conventional CT images, uncovering tumor phenotypic characteristics that are otherwise visually undetectable [5]. Rapid advances in machine learning algorithms and artificial intelligence (AI) have enabled the integration of radiomics data and clinical information, successfully creating accurate predictive models for various malignancies including lung, colorectal, and breast cancers [6,7]. Clinical studies have confirmed that CT-based radiomics not only effectively differentiates between benign and malignant lesions but also provides valuable references for individualized treatment strategies, playing an increasingly important role in clinical decision making [8,9].

Previous studies have widely applied radiomics techniques for classification and staging prediction in esophageal cancer [10,11]. Although relatively stable predictive models have been established, several limitations persist. First, the limited number of surgical cases included in studies may compromise model generalizability. Second, most existing models rely on conventional machine learning algorithms. Most importantly, these studies have exclusively focused on imaging features derived from the tumor region, potentially overlooking the broader physiological status of patients.

Muscle loss, particularly sarcopenia, has been increasingly recognized as a negative prognostic factor in various malignancies, including esophageal cancer [12,13]. Several studies have demonstrated that reduced skeletal muscle mass is associated with poor tolerance to neoadjuvant therapy, higher rates of postoperative complications, and inferior survival outcomes [14,15]. Postoperative esophageal cancer patients are particularly susceptible to muscle mass loss due to impaired nutritional intake [10,11]. Therefore, incorporating muscle-related imaging biomarkers—such as those extracted from the erector spinae and iliopsoas muscles—may provide additional prognostic insights beyond the tumor-centric features. These imaging indicators reflect systemic physiological conditions, including nutritional and functional status, which are critical to patient recovery and often influence treatment outcomes. By integrating these muscle features with conventional tumor features, we aim to enhance model performance and support more comprehensive, individualized clinical decision making.

This retrospective study included preoperative CT imaging data from 1066 patients undergoing surgery for esophageal cancer. Three sets of imaging features were systematically extracted: features from the esophageal region alone, combined esophageal and stomach regions, and the iliopsoas and erector spinae regions. Using traditional machine learning, deep learning, and multimodal fusion techniques, we constructed predictive models for postoperative pathological classification, T staging, and N staging. This paper is organized as follows: Section 2 includes clinical cohort establishment, image processing, and model construction; Section 3 includes the experimental results and model performance across different imaging modalities; Section 4 compares our findings with previous studies; and Section 5 offers a summary of key contributions and future research directions. This study particularly emphasizes the contribution of muscle imaging features and proposes a novel, integrative modeling framework for improving diagnostic accuracy and robustness in esophageal cancer. This study introduces a novel modeling strategy that integrates both tumor-specific and muscle-derived imaging features, an approach that has not been systematically investigated in previous radiomics research on esophageal cancer.

## 2. Methods and Materials

This section describes the data collection procedures, imaging processing workflow, and modeling strategies used to develop and evaluate the predictive models in this study.

### 2.1. Clinical Cohort Establishment

**Figure 1:**
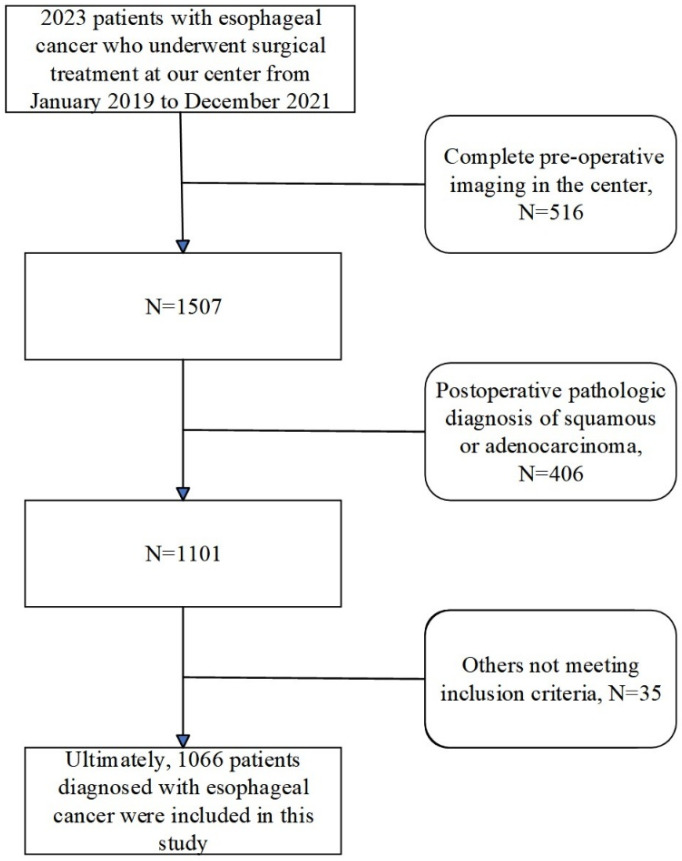
Clinical cohort establishment flowchart.

This study was formally approved by the Ethics Committee of the First Affiliated Hospital of Anhui Medical University (14 December 2023). We retrospectively enrolled 1066 patients who underwent radical esophagectomy for esophageal cancer at our center between December 2019 and December 2021. The detailed patient selection and enrollment processes are illustrated in Figure 1, while specific inclusion and exclusion criteria are provided in Supplementary Table S1. All included patients completed preoperative non-contrast CT scans and postoperative pathological assessments. The research team systematically collected imaging data, clinical baseline information, and pathological outcomes from each patient to construct a dedicated database. All endoscopic images in this study were obtained from the digestive endoscopy center of the First Affiliated Hospital of Anhui Medical University and were collected between 2019 and 2021. All cases of esophageal cancer were confirmed by postoperative pathological diagnosis, constituting a retrospective single-center study.

To develop predictive models, three critical pathological indicators of esophageal cancer were selected based on clinical significance and current research trends: pathological type (squamous cell carcinoma or adenocarcinoma), T-stage, and N-stage (lymph node metastasis number). Both T-stage and N-stage significantly impact treatment planning, as the T-stage reflects the tumor invasion depth, influencing surgical approaches and postoperative therapy, while the N-stage indicates lymph node metastasis extent, affecting the surgical scope and thoroughness of lymphadenectomy. Both indicators directly influence patient prognosis and therapeutic outcomes.

Notably, only patients with squamous cell carcinoma or adenocarcinoma were included in this study, excluding other rare cancer types. Accurate preoperative identification of pathological types, T-stage, and N-stage is essential for guiding perioperative chemotherapy and radiotherapy strategies. Improved preoperative prediction accuracy could facilitate more precise adjuvant therapy, thereby enhancing patient outcomes.

### 2.2. Imaging Data Collection and Processing

CT datasets used in this study were acquired preoperatively using a Philips Brilliance i CT 256 scanner (Philips Medical Systems Inc, Cleveland, OH, USA) as part of routine patient management. Images had a matrix resolution of 512 × 512 pixels and focused on thoracoabdominal regions, including the esophagus. To ensure comprehensive bilateral iliopsoas muscle imaging, preference was given to thoracoabdominal CT scans, with isolated chest CT scans utilized only if thoracoabdominal scans were unavailable.

**Figure 2:**
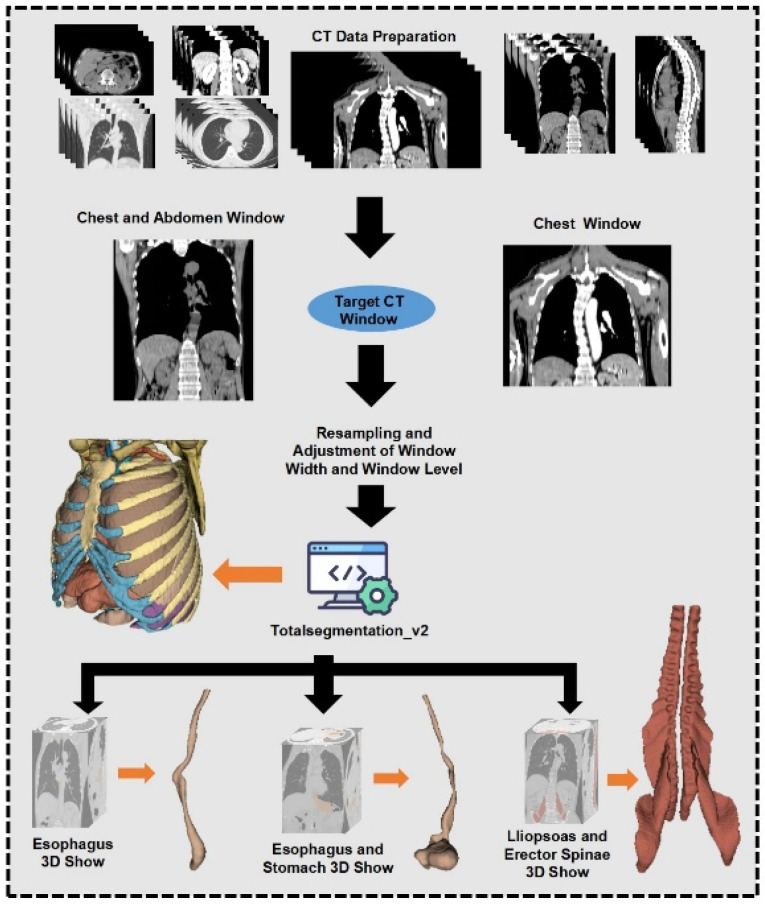
Image data processing and outlining process.

Original image slice thicknesses varied between 1.25 mm and 5 mm. To standardize image quality, voxel resolution was uniformly resampled to 3 mm, with window width (WW) set at 2000 and window level (WL) at −1000. Region of interest (ROI) segmentation was performed automatically using TotalSegmentation (V2.) software to create 3D ROI models of the esophagus, esophagus plus stomach, and associated muscle regions. Automated segmentations (V2.) were meticulously reviewed and manually corrected by two senior radiologists to meet the study’s stringent quality standards. All imaging data conformed to relevant European standards, detailed further in Supplementary Tables S2 and S3. Although a formal quantitative assessment of information loss was not performed, the resampling protocol was selected based on widely adopted standards in radiomics studies and visually confirmed to retain anatomical integrity during radiologist review. Figure 2 provides a detailed illustration of the image processing and segmentation workflow.

### 2.3. Radiomics Workflow

**Figure 3:**
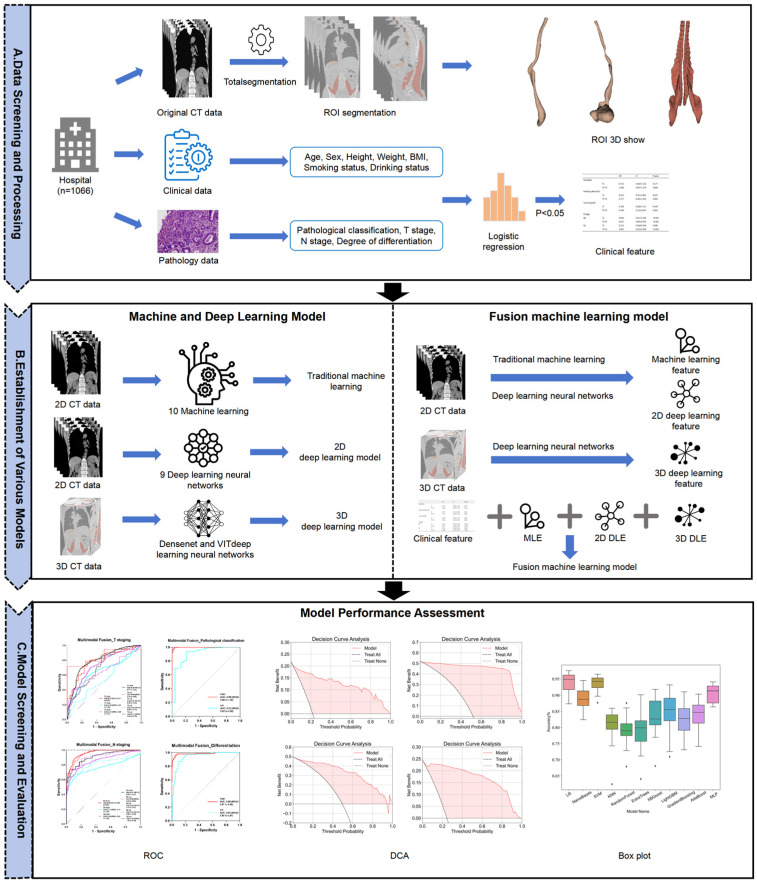
Flowchart of imaging analysis.

The radiomics analysis workflow used in this study is depicted in Figure 3. Two technical approaches were employed to construct radiomics diagnostic models: a deep learning approach using both 2D and 3D neural networks and a traditional machine learning approach involving feature extraction and various algorithms [16]. Model training and data processing were executed under a CUDA environment.

For traditional machine learning, the PyRadiomics (3.1.0) package in Python (3.9) was used for feature extraction from CT images covering the esophageal, esophageal plus stomach, and bilateral iliopsoas and erector spinae muscles [17,18]. Extracted features underwent rigorous screening using Least Absolute Shrinkage and Selection Operator (LASSO) regression to reduce redundancy and Spearman correlation analysis to eliminate highly correlated features (r > 0.9). The optimal feature subset was selected based on 5-fold cross-validation performance in the training set, with AUC serving as the primary evaluation metric. Data were randomly split into training (80%) and testing (20%) sets, balancing class distributions. Due to the notable class imbalance in the original dataset, especially for pathological subtypes and advanced staging categories, we manually balanced the validation set to ensure reliable performance assessment across all classes. The training set retained the original distribution to preserve real-world data characteristics. To mitigate the risk of overfitting, data augmentation techniques were applied during the training process. Eleven machine learning algorithms, logistic regression (LR), Naive Bayes, support vector machine (SVM), K-Nearest Neighbors (KNN), Random Forest (RF), ExtraTrees, XGBoost, LightGBM, GradientBoosting, AdaBoost, and multilayer perceptron (MLP), were applied [19,20,21]. Model performance was evaluated using 5-fold cross-validation, focusing on accuracy and AUC metrics.

The deep learning path employed 2D neural networks analyzing maximal cross-sectional ROI images and 3D neural networks using volumetric ROI data. Training lasted 100 epochs, utilizing 5-fold cross-validation with accuracy and AUC as primary metrics. Nine models from four neural network families (DenseNet121, DenseNet201, ResNet18, ResNet50, ResNet152, VGG13_bn, VGG19_bn, ViT, and SimpleViT) were evaluated, with the best-performing model selected per modality [22,23,24,25].

To compare diagnostic performance between esophageal, esophageal plus stomach, and muscle regions comprehensively, traditional machine learning and deep learning models underwent systematic comparative analysis. Statistical analyses of differences in AUC and accuracy were conducted to assess the predictive value of various imaging regions.

For radiomics feature fusion, multimodal features from traditional machine learning (models with accuracy and AUC > 0.5), 2D deep learning, and 3D deep learning were integrated. Deep learning features extracted from the final pooling layers were dimensionally reduced to 32 channels via Principal Component Analysis (PCA). Multimodal fusion models were developed for each pathological indicator with the imaging modalities included in Table 1.

### 2.4. Statistical Analysis and Machine Learning Tools

Statistical analyses were performed using SPSS 27.0. Continuous variables were presented as mean ± standard deviation (Mean ± SD), while categorical variables were described as frequency (*n*) and percentage (%). Chi-square tests were applied to categorical data, and independent sample *t*-tests or Mann–Whitney U-tests were selected for continuous variables according to data distribution. Univariate logistic regression assessed relationships between clinical variables and outcomes, followed by stepwise multivariate regression for significant variables.

Data preprocessing, feature extraction, and machine learning algorithm implementation were conducted using Python (3.9) libraries including NumPy, Pandas, Scikit-learn, and PyRadiomics. Deep learning models were developed and trained using the PyTorch (2.7.0) framework with GPU acceleration via CUDA. Computations were performed on high-performance hardware (Intel Core i9 processor, Intel Corporation, Santa Clara, CA, USA and NVIDIA GeForce RTX 4090 GPU, NVIDIA Corporation, Santa Clara, CA, USA).

Deep learning training employed mixed precision to optimize memory usage and distributed DataLoader for accelerated data input. Training utilized dynamic learning rate scheduling, Adam optimizer, data augmentation techniques, early stopping, and recorded training losses and validation metrics to identify optimal models.

## 3. Results

This section shows the experimental results and model performance across different imaging modalities.

### 3.1. Clinical Cohort Characteristics

This study enrolled 1066 patients who underwent surgical treatment for esophageal cancer. Clinical parameters collected included age, sex, height, weight, BMI, smoking history, and drinking history. The primary outcome variables analyzed were pathological type (squamous cell carcinoma or adenocarcinoma), T-stage, and N-stage (number of lymph node metastases). Detailed baseline clinical characteristics are presented in Table 2. Patient age ranged from 40 to 92 years, and BMI ranged from 14.88 to 39.13 kg/m^2^. Univariate and multivariate logistic regression analyses were conducted to examine relationships between clinical parameters and outcome variables. A stepwise regression model was applied in the multivariate analysis, with statistical significance defined as *p* < 0.05.

Regarding pathological types, univariate and multivariate analyses revealed significant correlations with T-stage and N-stage. Multivariate analysis demonstrated an OR of 2.827 (CI: 1.279–6.250, *p* = 0.01) for pathological type with T3–T4 stages and an OR of 2.894 (CI: 1.726–4.850, *p* < 0.001) with N2-stage. No significant relationships were identified between pathological types and age, sex, height, weight, BMI, or smoking or drinking history (*p* > 0.05). When analyzing the T-stage, significant correlations were found with smoking history, pathological type, and N-stage. Multivariate analysis indicated an OR of 0.622 (CI: 0.411–0.942, *p* = 0.025) for T2-stage with smoking history and an OR of 0.296 (CI: 0.135–0.647, *p* = 0.002) for T3–T4 stages with pathological type. T-stage and N-stage showed a statistically significant correlation (*p* < 0.05), reflecting clinical experience. However, age, sex, height, weight, BMI, and drinking history did not significantly correlate with T-stage (*p* > 0.05). For the N-stage, significant associations were observed with pathological type and T-stage. Multivariate analysis indicated an OR of 0.384 (CI: 0.231–0.639, *p* < 0.001) for N2–N3 stages with pathological type. Similarly, the T-stage demonstrated statistically significant correlations with the N-stage (*p* < 0.001). No significant associations were identified between the N-stage and other clinical characteristics (*p* > 0.05).

Overall, the results indicated significant relationships among pathological type, T-stage, and N-stage of esophageal cancer, with limited influence from basic clinical characteristics (such as age, sex, and BMI). Smoking history affected T-stage moderately but was insignificant in subsequent multimodal fusion analyses. Detailed univariate and multivariate logistic regression results are provided in Supplementary Tables S4–S9, respectively.

### 3.2. Differential Diagnostic Models for Squamous Cell Carcinoma and Adenocarcinoma

Based on imaging modalities from the esophageal phase, esophageal plus stomach phase, and muscle phase, 33 diagnostic models were constructed using 11 machine learning algorithms. Comparative analysis revealed no significant differences in diagnostic performance or stability between the models based on muscle image features and those based on esophageal or esophageal plus stomach features (*p* > 0.05). The results highlight the importance of muscle features in distinguishing esophageal squamous cell carcinoma from adenocarcinoma. Detailed comparative results are summarized in Table 3, Table 4 and Table 5, with additional performance metrics available in Supplementary Table S10.

Although no statistically significant differences were observed among the three imaging modalities in terms of accuracy and AUC (all *p* > 0.05), several trends can be noted. First, radiomics models based on esophageal-phase features achieved the highest AUC (0.78 ± 0.09) in the training set, suggesting better discriminatory power for histological classification. However, in the test set, performance metrics declined across all modalities, possibly due to data imbalance or generalization limitations. Interestingly, the muscle-phase models yielded comparable AUCs despite not directly targeting tumor morphology, indicating their potential as complementary features. The absence of statistical significance (e.g., *p* = 0.06 in Table 3 AUC comparison) may be attributed to sample imbalance, especially between squamous cell carcinoma and adenocarcinoma. These findings support the utility of both tumor-specific and systemic muscle features for differential diagnosis, though further validation in balanced or prospective datasets is warranted.

**Figure 4:**
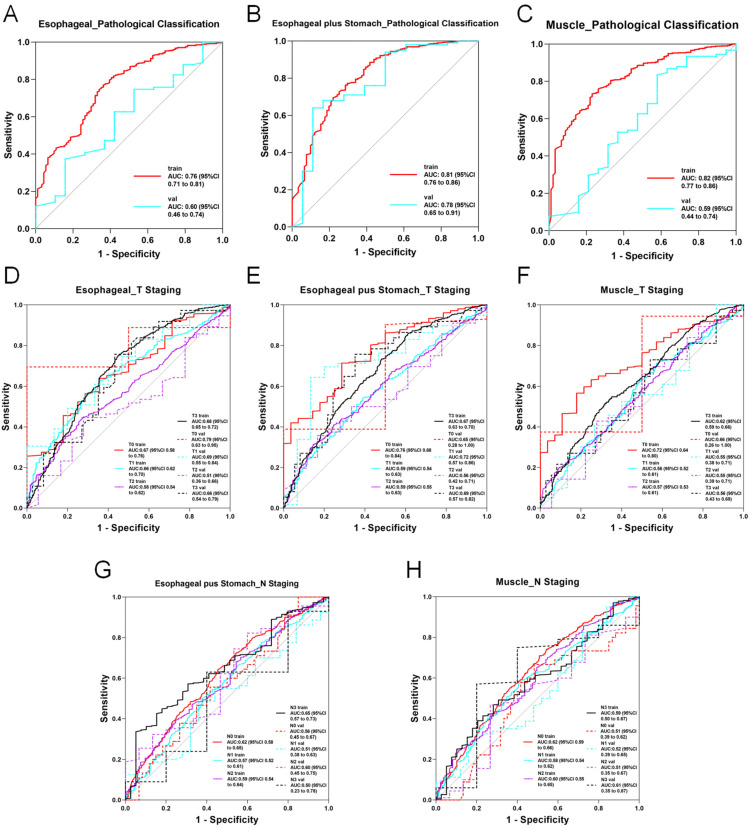
ROC curve of the optimal modality in 2D deep learning. (**A**) AUC curve of the optimal model for predicting esophageal cancer pathological classification based on esophageal imaging data. (**B**) AUC curve of the optimal model for predicting esophageal cancer pathological classification based on esophageal plus stomach imaging data. (**C**) AUC curve of the optimal model for predicting esophageal cancer pathological classification based on muscle imaging data. (**D**) AUC curve of the optimal model for predicting esophageal cancer T staging based on esophageal imaging data. (**E**) AUC curve of the optimal model for predicting esophageal cancer T staging based on esophageal plus stomach imaging data. (**F**) AUC curve of the optimal model for predicting esophageal cancer T staging based on muscle imaging data. (**G**) AUC curve of the optimal model for predicting esophageal cancer N staging based on esophageal plus stomach imaging data. (**H**) AUC curve of the optimal model for predicting esophageal cancer N staging based on muscle imaging data.

In the 2D deep learning pathway, 2D slices from each imaging modality were analyzed using nine neural network models with transfer learning. For features from the esophageal phase, DenseNet201 achieved the highest performance, with accuracy (ACC) and area under the curve (AUC) of 0.753 and 0.763 in the training set and 0.700 and 0.626 in the test set, respectively. For the esophagus plus stomach phase, DenseNet201 again showed optimal performance, reaching an ACC of 0.855 and AUC of 0.828 in the training set and 0.856 and 0.802 in the test set. Muscle-phase features using DenseNet201 resulted in training ACC and AUC of 0.737 and 0.843 and test ACC and AUC of 0.755 and 0.607, respectively. Several models demonstrated excellent predictive abilities, with ACC and AUC values above 0.8. Detailed 2D deep learning results are provided in Supplementary Table S11, with ROC curves for the optimal models presented in Figure 4A–C.

**Figure 5:**
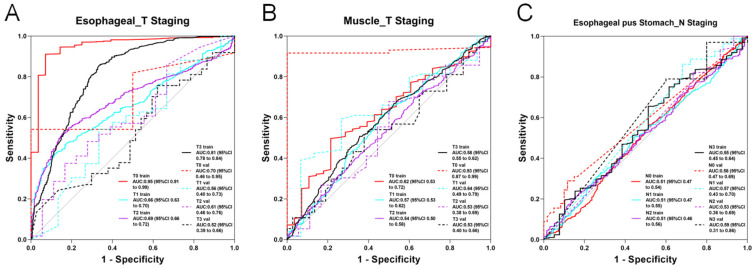
ROC curve of the optimal modality in 3D deep learning. (**A**) AUC curve of the optimal model for predicting esophageal cancer T staging based on esophageal imaging data. (**B**) AUC curve of the optimal model for predicting esophageal cancer T staging based on muscle imaging data. (**C**) AUC curve of the optimal model for predicting esophageal cancer N staging based on esophageal plus stomach imaging data.

In contrast, the performance of the 3D deep learning models was below expectations. When 3D volumetric data from each imaging modality were input into two neural network models, the results showed limited diagnostic capability, with many ACC and AUC values below 0.5. Consequently, 3D deep learning models were excluded from subsequent multimodal fusion analyses. Other 3D models are shown in Figure 5. Detailed 3D deep learning results are documented in Supplementary Table S12.

**Figure 6:**
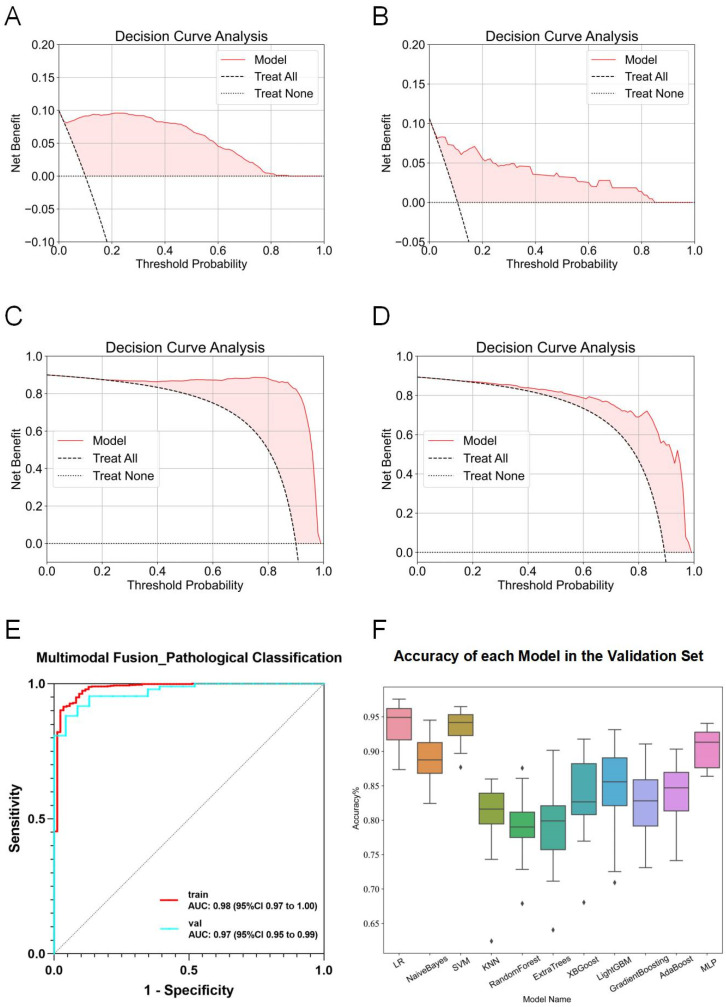
Detailed results of the fusion model for predicting esophageal cancer pathological classification. (**A**) DCA curve of the model predicting esophageal adenocarcinoma in the training set. (**B**) DCA curve of the model predicting esophageal adenocarcinoma in the validation set. (**C**) DCA curve of the model predicting esophageal squamous cell carcinoma in the training set. (**D**) DCA curve of the model predicting esophageal squamous cell carcinoma in the validation set. (**E**) ROC curve of the fusion model for predicting esophageal cancer pathological classification. (**F**) ACC of each fusion model in the validation set.

In multimodal fusion analyses, features from six imaging modalities (traditional machine learning and 2D deep learning for the esophageal, esophageal plus stomach, and muscle phases) were integrated and evaluated using 11 machine learning algorithms. Several fusion models exhibited exceptional performance, achieving ACC and AUC values above 0.9 in both training and test sets. LR consistently showed the highest performance, achieving an AUC of 0.98 (95% CI: 0.97–1.00) in the training set and 0.97 (95% CI: 0.95–0.99) in the validation set over 20 rounds of 5-fold cross-validation (Figure 6E). The box diagram is shown in Figure 6F. Decision curve analysis (DCA) further confirmed the clinical applicability and superior classification capabilities of the model for distinguishing esophageal squamous cell carcinoma from adenocarcinoma (Figure 6A–D).

### 3.3. Construction of T-Stage Diagnostic Models Based on Radiomics

To establish T-stage diagnostic models for esophageal cancer, initial analysis using traditional machine learning showed no significant correlation between muscle-phase imaging features and T-stage. The esophageal plus stomach phase models also failed to demonstrate meaningful classification performance. Thus, only esophageal-phase imaging features were retained for the traditional machine learning analysis. However, models based solely on esophageal-phase features exhibited limited overall performance, with accuracy and AUC rarely exceeding 0.7 consistently. For instance, the RandomForest model reached an accuracy of 0.988 in the training set but decreased sharply to 0.611 in the validation set, indicating potential overfitting. Comparative analysis of the three modalities revealed that for T1-stage prediction, models based on esophageal features significantly outperformed those based on the esophageal plus stomach modality (0.78 ± 0.08 vs. 0.58 ± 0.05, *p* < 0.01), while no significant differences were observed between other modalities (*p* > 0.05). Comprehensive results are summarized in Table 6, with detailed accuracy and AUC statistics provided in Supplementary Table S10.

In the 2D deep learning pathway, ROI data from each modality were input into nine neural network models across four model families. The best-performing model using esophageal-phase features was ResNet152, achieving an ACC and AUC of 0.591 and 0.697 in the training set and 0.514 and 0.574 in the test set, respectively. Using esophagus plus stomach features, DenseNet201 showed superior performance, with training ACC and AUC of 0.673 and 0.838 and test ACC and AUC of 0.676 and 0.705. Muscle-phase features analyzed by ResNet152 resulted in ACC and AUC values of 0.706 and 0.842 for training and 0.784 and 0.597 for testing. Models based on esophagus plus stomach and muscle-phase features demonstrated relatively stronger diagnostic capabilities compared with the esophageal-phase model. Detailed 2D deep learning results are provided in Supplementary Table S11, and ROC curves for optimal models are shown in Figure 4D–F.

In the 3D deep learning analysis using ResNet152, diagnostically valuable models were constructed for esophageal and muscle-phase modalities. For the esophageal phase, ResNet152 achieved ACC and AUC values of 0.82 and 0.926 in training and 0.743 and 0.577 in testing. For muscle-phase features, ACC and AUC were 0.59 and 0.571 (training), and 0.622 and 0.641 (testing), respectively. Although 3D models displayed limited overall stability and predictive accuracy, some demonstrated potential utility in specific scenarios. ROC curves for these models are presented in Figure 5A,B, with further details provided in Supplementary Table S12.

**Figure 7:**
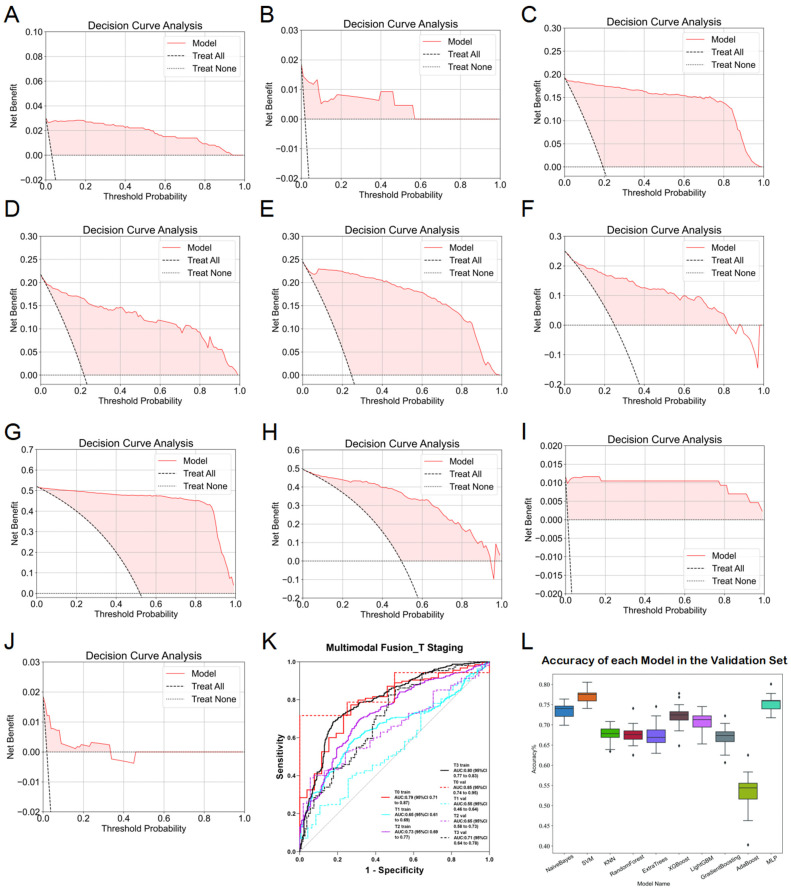
Detailed results of the fusion model for predicting esophageal cancer T staging. (**A**) DCA curve of the model predicting esophageal cancer Tis staging in the training set. (**B**) DCA curve of the model predicting esophageal cancer Tis staging in the validation set. (**C**) DCA curve of the model predicting esophageal cancer T1 staging in the training set. (**D**) DCA curve of the model predicting esophageal cancer T1 staging in the validation set. (**E**) DCA curve of the model predicting esophageal cancer T2 staging in the training set. (**F**) DCA curve of the model predicting esophageal cancer T2 staging in the validation set. (**G**) DCA curve of the model predicting esophageal cancer T3 staging in the training set. (**H**) DCA curve of the model predicting esophageal cancer T3 staging in the validation set. (**I**) DCA curve of the model predicting esophageal cancer T4 staging in the training set. (**J**) DCA curve of the model predicting esophageal cancer T4 staging in the validation set. (**K**) ROC curve of the fusion model for predicting esophageal cancer T staging. (**L**) ACC of each fusion model in the validation set.

During multimodal fusion analysis, seven-dimensional imaging features were combined, incorporating traditional machine learning esophageal-phase features, 2D deep learning features (ResNet152 for esophageal phase, DenseNet201 for esophagus plus stomach phase, and ResNet152 for muscle phase), and 3D deep learning features (ResNet152 for esophageal and muscle phases). Smoking history was initially considered but was excluded after regression analyses showed no significant correlation with T-stage. Multimodal features were evaluated using ten machine learning algorithms, several of which exhibited robust performance with ACC and AUC consistently above 0.7. Specifically, predictions for Tis-, T1-, T2-, and T3-stages achieved notable AUCs in both training and validation sets, with the highest performance observed in T3-stage predictions (training AUC = 0.80, validation AUC = 0.71). After 20 rounds of cross-validation, SVM models demonstrated the highest accuracy and stability (training ACC = 0.909, validation ACC = 0.907). However, due to limited data availability, T4-stage predictions remained inadequate, and ROC curves were not plotted. ROC curves for the final fusion diagnostic model are provided in Figure 7K, with DCA results detailed in Figure 7A–J.

### 3.4. Construction of N-Stage Diagnostic Models Based on Radiomics

Preoperative CT images of esophageal cancer patients were analyzed to construct diagnostic N-stage classification models. In traditional machine learning analysis, the KNN model based on esophageal plus stomach imaging features demonstrated the best performance, achieving accuracy rates of 0.614 and 0.648 in the training and validation sets, respectively. Comparative analysis among the three modalities (esophageal, esophageal plus stomach, and muscle) revealed modality-specific advantages: the esophageal modality outperformed muscle modality for predicting N0 (0.66 ± 0.09 vs. 0.54 ± 0.08, *p* = 0.005); esophageal plus stomach modality was superior for N1-stage prediction compared with esophageal modality (0.59 ± 0.13 vs. 0.46 ± 0.12, *p* = 0.02); muscle modality outperformed esophageal plus stomach modality for N2-stage prediction (0.63 ± 0.11 vs. 0.52 ± 0.11, *p* = 0.03); and esophageal modality performed better than esophageal plus stomach modality for N3-stage prediction (0.51 ± 0.15 vs. 0.31 ± 0.17, *p* = 0.01). Detailed comparative results are presented in Table 7, Table 8 and Table 9, with further accuracy and AUC statistics provided in Supplementary Table S10.

In the 2D deep learning pathway, both esophageal plus stomach and muscle modalities exhibited limited but meaningful diagnostic potential, particularly with the ResNet50 model. When utilizing esophageal-phase features, none of the nine models reached satisfactory accuracy or AUC. The best-performing model for the esophageal plus stomach phase (ResNet50) achieved ACC and AUC of 0.675 and 0.644 in the training set and 0.505 and 0.536 in the test set, respectively. For the muscle phase, ResNet50 reached an ACC and AUC of 0.584 and 0.583 in the training set and 0.581 and 0.517 in the test set. Detailed training outcomes and ROC curves for optimal 2D models are provided in Supplementary Table S11 and Figure 4G–H.

In the 3D deep learning analysis, the ResNet152 model displayed some diagnostic capability in the esophageal plus stomach modality, although accuracy and AUC remained moderate. For esophageal-phase features, ResNet152 showed training ACC and AUC of 0.51 and 0.523 with test ACC and AUC of 0.752 and 0.47. For the esophageal plus stomach phase, ResNet152 reached ACC and AUC of 0.511 and 0.512 in training and 0.724 and 0.568 in testing, respectively. Muscle-phase results were less impressive. Detailed results and ROC curves are available in Supplementary Table S12 and Figure 5C.

**Figure 8:**
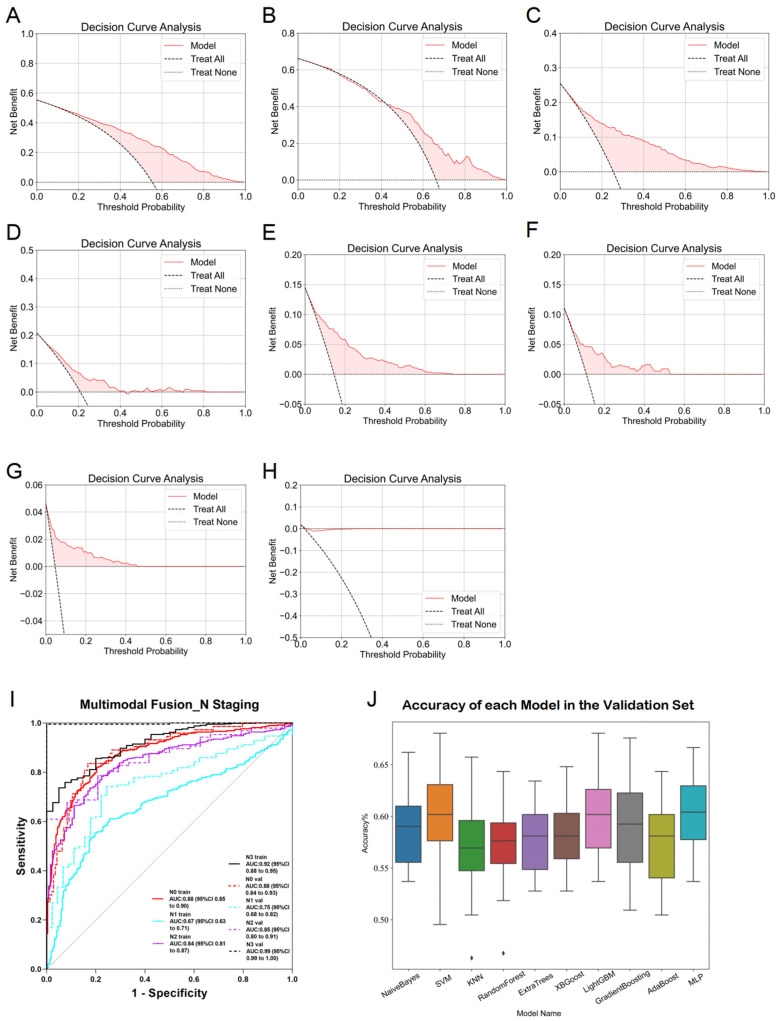
Detailed results of the fusion model for predicting esophageal cancer N staging. (**A**) DCA curve of the model predicting esophageal cancer N0 staging in the training set. (**B**) DCA curve of the model predicting esophageal cancer N0 staging in the validation set. (**C**) DCA curve of the model predicting esophageal cancer N1 staging in the training set. (**D**) DCA curve of the model predicting esophageal cancer N1 staging in the validation set. (**E**) DCA curve of the model predicting esophageal cancer N2 staging in the training set. (**F**) DCA curve of the model predicting esophageal cancer N2 staging in the validation set. (**G**) DCA curve of the model predicting esophageal cancer N3 staging in the training set. (**H**) DCA curve of the model predicting esophageal cancer N3 staging in the validation set. (**I**) ROC curve of the fusion model for predicting esophageal cancer N staging. (**J**) ACC of each fusion model in the validation set.

**Figure 9:**
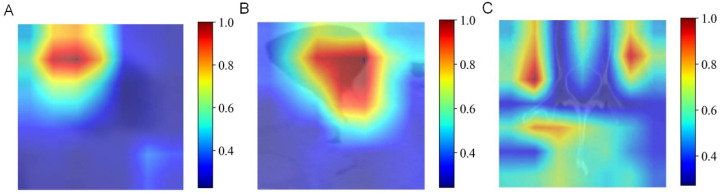
Heatmaps of the key regions identified by the AI model. (**A**) Esophageal phases. (**B**) Esophageal plus stomach phases. (**C**) Muscle phases.

In multimodal fusion analysis, imaging features from traditional machine learning (esophageal plus stomach), 2D deep learning (ResNet50 from esophageal plus stomach and muscle modalities), and 3D deep learning (ResNet152 from esophageal plus stomach modality) were integrated. Evaluations of these fused features using ten machine learning algorithms resulted in improved overall performance, consistently achieving ACC and AUC values around 0.6. Specifically, AUC values for predicting N0-, N1-, N2-, and N3-stages demonstrated significant improvements, with the highest performance seen in predicting the N3-stage (training AUC = 0.92, validation AUC = 0.99). However, caution regarding potential overfitting in N3-stage predictions is advised and further discussed in subsequent sections. Comparative analysis (Figure 8J) indicated that the MLP algorithm yielded optimal fusion model performance, with training and validation accuracies of 0.704 and 0.685, respectively. Detailed accuracy results post-fusion for the validation set are presented in Figure 8I, and DCA results are shown in Figure 8A–H. Finally, heatmaps are provided to visualize the key regions identified by the model, enhancing its interpretability in Figure 9.

## 4. Discussion

This section compares our findings with previous studies and discusses the research implications and limitations.

In recent years, radiomics analysis has become a powerful tool for predicting pathological types and staging in esophageal cancer. By extracting high-dimensional quantitative features from imaging data such as CT, radiomics comprehensively reveals tumor heterogeneity, morphology, and metabolic activity [11]. In this study, predictive models for esophageal cancer pathology type and staging were developed based on CT imaging features of the esophageal, esophageal plus stomach, and bilateral iliopsoas and erector spinae muscles, employing traditional machine learning, deep learning, and multimodal fusion techniques. Importantly, this study is the first to incorporate imaging features of the iliopsoas and erector spinae muscles into the predictive analysis of esophageal cancer pathology and staging. Previous research has demonstrated the prognostic significance of muscle quality in cancer outcomes, and our findings further support this perspective [10].

Radiomics using CT imaging has shown considerable potential for diagnosing and classifying esophageal cancer. By extracting extensive quantitative features from CT images and applying machine learning algorithms, radiomics effectively distinguishes between esophageal squamous cell carcinoma and adenocarcinoma. Previous studies have reported that CT-based radiomics models achieve high accuracy and stability in diagnosing esophageal cancer subtypes Table 10. For example, Du, K.P. et al. constructed classification models using multi-level imaging features, achieving an AUC exceeding 90%, significantly outperforming conventional diagnostic methods [10]. Other studies have also validated the value of radiomics features in predicting tumor staging and prognosis, enhancing their clinical applicability [26]. In the study, a high-accuracy diagnostic model for distinguishing squamous cell carcinoma from adenocarcinoma was developed by integrating multimodal features from traditional machine learning and deep learning analyses of CT images. This model achieved an AUC of 0.99 (95% CI: 0.99–1.00) in the training set and 0.90 (95% CI: 0.83–0.98) in the validation set, surpassing previous studies. Overall, our CT-based radiomics diagnostic model significantly improves the efficiency of esophageal cancer subtype identification and provides critical imaging support for personalized treatment planning.

Accurate T-stage assessment plays a pivotal role in esophageal cancer treatment decisions, as it directly affects treatment strategies. For instance, Tis (carcinoma in situ) and T1 tumors are typically managed by endoscopic resection or local surgical procedures, while T2 or more advanced tumors may require combined chemoradiotherapy or radical surgery. T3 tumors often necessitate neoadjuvant treatments (e.g., chemoradiotherapy) to improve resectability and survival due to potential infiltration into adjacent tissues. T4 tumors, which invade neighboring organs or structures, are generally deemed unresectable, with treatment focused primarily on palliative care. CT-based radiomics holds significant promise in this context. By extracting quantitative imaging features combined with advanced machine learning techniques, radiomics provides novel opportunities for precise T-stage prediction in esophageal cancer. Prior studies have demonstrated high accuracy and consistency of radiomics-based predictive models for T-stage evaluation. For example, Lei, X. et al. and Yang, M. et al. developed models using multidimensional imaging features achieving AUC values up to 0.85, significantly outperforming traditional radiological assessment [27,28]. Multicenter studies have further validated the robustness of radiomics features, laying a solid foundation for clinical translation [30]. In this study, we utilized multimodal fusion methods to construct T-stage prediction models aimed at improving staging accuracy and supporting personalized therapeutic decisions. While the models demonstrated high predictive performance for the T2- and T3-stages, predictions for the T1- and T4-stages were less accurate, somewhat reducing overall performance. This discrepancy may result from limited sample sizes in the T1- and T4-stages, highlighting critical challenges in applying pathology classification models in real clinical settings. Overall, our research confirms the potential of radiomics for T-stage prediction in esophageal cancer while emphasizing the importance of model generalization and clinical applicability during model development.

The N-stage significantly impacts therapeutic decisions in patients with esophageal cancer, and accurately predicting lymph node metastasis is crucial for personalized cancer treatment. Radiomics technology provides novel tools for predicting lymph node metastasis by extracting high-dimensional quantitative features from medical images. Numerous studies have developed radiomics-based models for predicting lymph node metastasis in esophageal cancer, some demonstrating exceptional performance and potential for clinical implementation. A meta-analysis incorporating 12 radiomics studies showed average training and validation set AUCs of 0.87 and 0.85, respectively, underscoring the strong diagnostic efficacy of radiomics [29]. However, previous studies have certain limitations. First, most studies had small sample sizes, typically including around 200 patients or fewer, limiting model optimization and generalizability. Additionally, many simplified data by merging N1- and N2-stages or treating lymph node status as a binary classification, thus neglecting independent prediction capabilities for each N-stage, diminishing clinical relevance. In this study, we developed diagnostic models for esophageal cancer N staging using a large sample cohort and thoroughly assessed predictive performance for each N-stage. Our models achieved excellent predictive performance (AUC > 0.85) across most stages, except N1. Notably, high predictive performance for N3 in validation could reflect limited sample sizes within this group. Additionally, our study uniquely incorporated muscle imaging features into the N-stage prediction for esophageal cancer, further enhancing diagnostic efficacy through multimodal fusion. Compared with pathological subtypes and T staging, the prediction of N staging remains more challenging. This may be attributed to the limited spatial resolution of CT images in identifying metastatic lymph nodes and the subtle imaging manifestations of N2-stage involvement. In addition, the annotation of nodal staging is often subject to interobserver variability and lacks precise radiologic correlations, which may affect model training. Therefore, despite the integration of multimodal features, the MLP model achieved moderate accuracy. Further studies incorporating functional imaging (e.g., PET-CT), radiogenomic data, or attention-based neural networks may help improve N staging prediction performance.

To the best of our knowledge, prior studies have not incorporated muscle imaging features into radiomics models for esophageal cancer. One possible reason is that traditional radiomics research has primarily focused on tumor-centric analysis, where only the primary lesion and its immediate surroundings are considered informative. Additionally, muscle imaging features require precise segmentation of regions beyond the tumor itself, which introduces technical complexity and necessitates standardized protocols for feature extraction. The potential prognostic role of skeletal muscle in oncology has only recently gained attention, with emerging evidence linking sarcopenia to adverse outcomes in various malignancies. Our study is among the first to translate this concept into radiomics modeling for esophageal cancer, highlighting the value of systemic physiological indicators beyond local tumor characteristics.

Overall, this research affirms radiomics’s potential for predicting N-stage in esophageal cancer and identifies key areas for improving model performance. Incorporating muscle imaging features not only expands radiomics applications but also provides new insights into intrinsic physiological correlations. This study achieved several breakthroughs in radiomics and medical imaging by integrating multimodal imaging data, including features from the esophagus, esophagus plus stomach, and bilateral iliopsoas and erector spinae muscles, significantly improving diagnostic accuracy. Compared with traditional radiomics research, our study provided deeper insights into the imaging data, offering new perspectives for quantifying tumor heterogeneity. Importantly, our innovative inclusion of muscle imaging features addresses a critical research gap. By combining features from muscles, esophagus, and esophagus plus stomach, our multimodal fusion models significantly enhanced accuracy in predicting pathological type and stage. The results confirmed the value of muscle imaging features in predicting pathological outcomes, revealing potential associations between muscle characteristics, tumor staging, and lymph node metastasis. These findings provide additional evidence linking muscle quality to cancer prognosis, broadening radiomics’s scope in oncological research. Leveraging CT imaging and advanced machine learning, our models improved diagnostic accuracy, facilitating early detection and potentially improving patient outcomes. The innovative methods applied in feature selection and validation set new standards for future studies, promoting precision medicine’s personalized approach.

Despite significant achievements, our study has limitations. First, we relied on automated segmentation tools for quantifying muscle quality, which, despite rigorous quality control checks and radiologist consensus, may introduce variability. Future research should validate these automated methods’ accuracy and reliability in clinical settings. Second, our data derived from a single center, which, despite encompassing a large esophageal cancer database, may limit generalizability due to a lack of multicenter validation. To address this, future studies should integrate radiomics with molecular mechanisms and incorporate multicenter clinical datasets to comprehensively explore diagnostic and prognostic challenges in esophageal cancer, thereby providing stronger scientific evidence for clinical treatment. At this stage, our study has undergone only internal validation (including cross-validation and an independent test set) and lacks external validation. And this is a single-center retrospective study with the following limitations: no multicenter external validation, potentially limiting generalizability; variable image quality that may affect model performance; and lack of human–AI comparison, leaving clinical relevance unassessed. To enhance the model’s generalizability and clinical applicability, future work will involve multicenter data and clinician comparison.

## 5. Conclusions

This study established predictive models for pathological classification, T staging, and N staging of esophageal cancer based on CT imaging data from 1066 patients. Comparative analyses of machine learning models constructed from esophageal, esophageal plus stomach, and muscle imaging modalities revealed that muscle imaging features contributed similarly to diagnostic accuracy as esophageal-phase imaging. The multimodal fusion models consistently demonstrated superior performance across all three pathological outcomes compared with single-modality models. Despite limitations related to single-center data potentially affecting model generalizability and the accuracy of automatic segmentation impacting feature extraction precision, our multimodal fusion radiomics model showed promising clinical application potential through extensive data training and validation. Future research should focus on multicenter clinical studies to validate model generalizability and optimize automatic segmentation algorithms to enhance feature extraction accuracy.

## Figures and Tables

**Table 1:** Statistical table of multimodal fusion models.

	Machine Learning	2D Deep Learning	3D Deep Learning	Algorithm
Pathological classification	Esophageal features Esophageal plus stomach features Muscle feature	Esophageal_densenet201 Esophageal plus stomach_densenet201 Muscle_densenet201	-	LR
T staging	Esophageal features	Esophageal_resnet152 Esophageal plus stomach_densenet201 Muscle_resnet152	Esophageal_resnet152 Muscle_resnet152	SVM
N staging	Esophageal plus stomach features	Esophageal plus stomach_resnet50 Muscle_resnet50	Esophageal plus stomach_resnet152	MLP

**Table 2:** Characteristics of the clinical population.

Characteristics	All	Mean + SD/*n* (%)
Age	1066	66.58 ± 8.08
BMI	1066	22.33 ± 3.08
Sex	1066	
Male		871 (81.71%)
Female		195 (18.29%)
Smoking status	1066	
No		681 (63.88%)
Yes		385 (36.12%)
Drinking status	1066	
No		749 (70.26%)
Yes		317 (29.74%)
Pathological classification	1066	
Squamous carcinoma		956 (89.68%)
Adenocarcinoma		110 (10.32%)
T staging	1066	
Tis		30 (2.81%)
T1		210 (19.70%)
T2		260 (24.39%)
T3		552 (51.78%)
T4		14 (1.31%)
N staging	1066	
N0		610 (57.22%)
N1		263 (24.67%)
N2		149 (13.98%)
N3		44 (4.13%)

**Table 3:** Comparative analysis of the predictive efficacy of esophageal phase versus muscle phase when constructing a predictive model for pathological classification of esophageal cancer.

Model Classification	Esophageal Phase	Muscle Phase	*p*-Value
Training Set			
ACC	0.63 ± 0.21	0.61 ± 0.25	0.72
AUC	0.78 ± 0.09	0.67 ± 0.14	0.06
Test Set			
ACC	0.63 ± 0.21	0.55 ± 0.21	0.42
AUC	0.66 ± 0.05	0.62 ± 0.12	0.84

**Table 4:** Comparative analysis of the predictive efficacy of esophageal phase versus esophageal plus stomach phase when constructing a pathological classification prediction model for esophageal cancer.

Model Classification	Esophageal Phase	Esophagus Plus Stomach Phase	*p*-Value
Training Set			
ACC	0.63 ± 0.21	0.59 ± 0.25	0.92
AUC	0.78 ± 0.09	0.70 ± 0.21	0.26
Test Set			
ACC	0.63 ± 0.21	0.68 ± 0.15	0.53
AUC	0.66 ± 0.05	0.60 ± 0.10	0.12

**Table 5:** Comparative analysis of the predictive efficacy of esophageal plus stomach phases versus muscle phases in constructing a predictive model for pathological classification of esophageal cancer.

Model Classification	Esophagus Plus Stomach Phase	Muscle Phase	*p*-Value
Training Set			
ACC	0.59 ± 0.25	0.61 ± 0.25	0.89
AUC	0.70 ± 0.21	0.67 ± 0.14	0.77
Test Set			
ACC	0.68 ± 0.15	0.55 ± 0.21	0.14
AUC	0.60 ± 0.10	0.62 ± 0.12	0.31

**Table 6:** Comparative analysis of the predictive efficacy of esophageal phase versus esophageal plus stomach phase when constructing a predictive model for T staging of esophageal cancer.

Model Classification	Esophageal Phase	Esophagus Plus Stomach Phase	*p*-Value
T Staging			
Train ACC	0.71 ± 0.19	0.64 ± 0.19	0.31
Test ACC	0.56 ± 0.06	0.53 ± 0.02	0.12
AUC			
T0	0.48 ± 0.19	0.45 ± 0.13	0.65
T1	0.78 ± 0.08	0.58 ± 0.05	<0.01
T2	0.61 ± 0.07	0.62 ± 0.05	0.79
T3	0.64 ± 0.03	0.63 ± 0.07	0.89
T4	0.32 ± 0.28	0.40 ± 0.35	0.56

**Table 7:** Comparative analysis of the predictive efficacy of esophageal phase versus muscle phase when constructing a predictive model for N staging esophageal cancer.

Model Classification	Esophageal Phase	Muscle Phase	*p*-Value
**N Staging**			
Train ACC	0.69 ± 0.17	0.68 ± 0.17	0.7
Test ACC	0.59 ± 0.03	0.56 ± 0.04	0.53
**AUC**			
N0	0.66 ± 0.09	0.54 ± 0.08	0.005
N1	0.46 ± 0.12	0.51 ± 0.07	0.231
N2	0.59 ± 0.12	0.63 ± 0.11	0.380
N3	0.51 ± 0.15	0.39 ± 0.13	0.08

**Table 8:** Comparative analysis of the predictive efficacy of esophageal phase versus esophageal plus stomach phase when constructing a predictive model for N staging of esophageal cancer.

Model Classification	Esophageal Phase	Esophagus Plus Stomach Phase	*p*-Value
**N Staging**			
Train ACC	0.69 ± 0.17	0.68 ± 0.17	0.64
Test ACC	0.59 ± 0.03	0.58 ± 0.02	0.29
**AUC**			
N0	0.66 ± 0.09	0.59 ± 0.09	0.09
N1	0.46 ± 0.12	0.59 ± 0.13	0.02
N2	0.59 ± 0.12	0.52 ± 0.11	0.20
N3	0.51 ± 0.15	0.31 ± 0.17	0.01

**Table 9:** Comparative analysis of predictive efficacy of esophageal plus stomach versus muscle phases in constructing predictive models for N staging of esophageal cancer.

Model Classification	Esophagus Plus Stomach Phase	Muscle Phase	*p*-Value
**N Staging**			
Train ACC	0.68 ± 0.17	0.68 ± 0.17	0.96
Test ACC	0.59 ± 0.03	0.56 ± 0.04	0.09
**AUC**			
N0	0.59 ± 0.09	0.54 ± 0.08	0.20
N1	0.59 ± 0.13	0.51 ± 0.07	0.10
N2	0.52 ± 0.11	0.63 ± 0.11	0.03
N3	0.31 ± 0.17	0.39 ± 0.13	0.26

**Table 10:** Comparative results between this study and previous studies.

Research	Number of Population	Target	AUC	Accuracy	Sensitivity	Specificity
Du, K.P. et al. [10]	260	Pathological Subtype	0.904	0.841	0.802	0.879
Lei, X. et al. [27]	100	T-stage	0.850	-	-	
Yang, M. et al. [28]	116	T-stage	0.860	-	0.77	0.87
Jannatdoust, P. et al. [29]	-	N-stage	0.870	-	0.787	0.818
This study	1066	Pathological Subtype	0.980	0.900	-	-
		T-stage	0.800	0.900	-	-
		N-stage	0.920	-	-	-

## Data Availability

The datasets and CT images used and analyzed during the current study are available from the corresponding author on reasonable request due to patient privacy issues.

## Supplementary Materials

The following supporting information can be downloaded at: https://www.mdpi.com/article/10.3390/diagnostics15141730/s1, Table S1: Specific inclusion and exclusion criteria; Table S2: CheckList for evaluation of radiomics; Table S3: Metrics; Table S4: Correlation between clinical characteristics and pathological classification of esophageal cancer by univariate logistic regression analysis; Table S5: Correlation between clinical characteristics and T staging of esophageal cancer by univariate logistic regression analysis; Table S6: Correlation between clinical characteristics and N staging of esophageal cancer by univariate logistic regression analysis; Table S7: Correlation between clinical characteristics and pathological classification of esophageal cancer by multivariate logistic regression analysis; Table S8: Correlation between clinical characteristics and T staging of esophageal cancer by multivariate logistic regression analysis; Table S9: Correlation between clinical characteristics and N staging of esophageal cancer by multivariate logistic regression analysis; Table S10: Detailed predictive efficacy of each model based on traditional machine learning methods; Table S11: Detailed predictive efficacy of each model based on 2d deep learning approach; Table S12: Detailed predicted efficacy of each model based on 3d deep learning approach. References [31,32] are cited in Supplementary Materials.

## Abbreviations

EC	Esophageal cancer
AI	Artificial intelligence
CT	Computed tomography
SVM	Support vector machine
MLP	Multilayer perceptron
BMI	Body mass index
ROC	Receiver Operating Characteristic Curve
AUC	Area under the curve
DCA	Decision curve analysis
CNN	Convolutional Neural Network
ROI	Region of interest
ResNet	Residual Network
DenseNet	Densely Connected Convolutional Network
VGG	Visual Geometry Group
RF	Random Forest
KNN	K-Nearest Neighbors
XGBoost	eXtreme Gradient Boosting
LightGBM	Light Gradient Boosting Machine
PCA	Principal Component Analysis
LR	Logistic regression
