# Supplementary material for: Machine Learning and Deep Learning Hybrid Approach Based on Muscle Imaging Features for Diagnosis of Esophageal Cancer
Source: Diagnostics (Basel). 2025 Jul 8;15(14):1730. doi: 10.3390/diagnostics15141730 (PMC12293794; doi:10.3390/diagnostics15141730)
Supplement: Supplementary file 1 [file diagnostics-15-01730-s001.zip › Supplementary Table S1.pdf]

Inclusion Criteria

1. Age > 18 years, no gender restriction;
2. Clear surgical indication and Eastern Cooperative Oncology Group (ECOG) performance status of 0-1;
3. Clinically judged to be able to tolerate surgical treatment with normal bone marrow, liver, kidney, heart, lung, and nervous system function;
4. No active autoimmune disease, no active hepatitis B, no HIV infection;
5. This study included patients who had received preoperative or postoperative adjuvant therapy;
6. Patients undergoing surgical treatment at our hospital with postoperative pathology confirming esophageal squamous cell carcinoma or adenocarcinoma, and having complete preoperative imaging data from our hospital;
7. Voluntarily participate and cooperate with the study, including but not limited to complying with treatment and follow-up, cooperating with the researchers in data collection, not seeking other treatments, and signing an informed consent form.

Exclusion Criteria

1. Presence of surgical contraindications, or evidence of distant metastasis at the time of diagnosis (e.g., brain, bone, liver metastasis);
2. Poor cardiopulmonary function that prevents tolerance of surgical treatment;
3. History of other malignant tumors;
4. Coexisting myocardial infarction, cerebral infarction, or other thromboembolic diseases that require surgical treatment;
5. Severe infection;
6. Postoperative pathology revealing neuroendocrine carcinoma, signet-ring cell carcinoma, or other types of esophageal cancer; missing preoperative imaging data or imaging performed outside the hospital;
7. Pregnant or breastfeeding women, or women planning to conceive;
8. Severe mental illness or other conditions that, in the judgment of the researcher, make participation in the study inappropriate.
