# Supplementary material for: Machine Learning and Deep Learning Hybrid Approach Based on Muscle Imaging Features for Diagnosis of Esophageal Cancer
Source: Diagnostics (Basel). 2025 Jul 8;15(14):1730. doi: 10.3390/diagnostics15141730 (PMC12293794; doi:10.3390/diagnostics15141730)
Supplement: Supplementary file 1 [file diagnostics-15-01730-s001.zip › Supplementary Table S10.pdf]

	Model_name	ACC	AUC	95% CI	Sensitivity	Specificity	PPV	NPV	Precision	Recall	F1	Threshold	Task
Esophageal_Pathological Classification													
	LR	0.69	0.659	0.5975 - 0.7209	0.704	0.557	0.94	0.159	0.94	0.704	0.805	0.898	label-train
	LR	0.745	0.694	0.5752 - 0.8135	0.762	0.645	0.928	0.312	0.928	0.762	0.837	0.889	label-test
	NaiveBayes	0.549	0.663	0.6034 - 0.7219	0.527	0.759	0.956	0.14	0.956	0.527	0.68	0.913	label-train
	NaiveBayes	0.833	0.723	0.6069 - 0.8400	0.876	0.581	0.926	0.439	0.926	0.876	0.9	0.859	label-test
	SVM	0.867	0.826	0.7799 - 0.8725	0.898	0.557	0.953	0.355	0.953	0.898	0.924	0.908	label-train
	SVM	0.435	0.529	0.4286 - 0.6291	0.384	0.742	0.899	0.168	0.899	0.384	0.538	0.908	label-test
	KNN	0.092	0.866	0.8428 - 0.8883	0	1	0	0.092	0	0	NaN	1	label-train
	KNN	0.144	0.622	0.5266 - 0.7167	0	1	0	0.144	0	0	NaN	1	label-test
	RandomForest	0.771	0.877	0.8398 - 0.9132	0.762	0.861	0.982	0.268	0.982	0.762	0.858	0.897	label-train
	RandomForest	0.685	0.676	0.5632 - 0.7886	0.686	0.677	0.927	0.266	0.927	0.686	0.789	0.904	label-test
	ExtraTrees	0.642	0.83	0.7883 - 0.8707	0.617	0.886	0.982	0.189	0.982	0.617	0.758	0.905	label-train
	ExtraTrees	0.815	0.692	0.5785 - 0.8063	0.87	0.484	0.91	0.385	0.91	0.87	0.89	0.889	label-test

	XGBoost	0.789	0.753	0.6979 - 0.8076	0.815	0.532	0.945	0.225	0.945	0.815	0.875	0.84	label-train
	XGBoost	0.792	0.661	0.5530 - 0.7683	0.849	0.452	0.902	0.333	0.902	0.849	0.875	0.84	label-test
	LightGBM	0.754	0.888	0.8556 - 0.9201	0.742	0.873	0.983	0.255	0.983	0.742	0.846	0.884	label-train
	LightGBM	0.731	0.661	0.5523 - 0.7691	0.757	0.581	0.915	0.286	0.915	0.757	0.828	0.881	label-test
	GradientBoosting	0.622	0.813	0.7665 - 0.8589	0.6	0.835	0.973	0.174	0.973	0.6	0.742	0.902	label-train
	GradientBoosting	0.63	0.702	0.6002 - 0.8036	0.616	0.71	0.927	0.237	0.927	0.616	0.74	0.902	label-test
	AdaBoost	0.588	0.756	0.7112 - 0.8003	0.563	0.835	0.971	0.162	0.971	0.563	0.713	0.559	label-train
	AdaBoost	0.579	0.633	0.5335 - 0.7317	0.557	0.71	0.92	0.212	0.92	0.557	0.694	0.564	label-test
	MLP	0.543	0.622	0.5633 - 0.6816	0.527	0.696	0.945	0.129	0.945	0.527	0.677	0.903	label-train
	MLP	0.505	0.664	0.5575 - 0.7708	0.449	0.839	0.943	0.203	0.943	0.449	0.608	0.909	label-test
Esophageal plus Stomach_Pathological Classification													
	LR	0.535	0.602	0.5380 - 0.6654	0.523	0.651	0.933	0.127	0.933	0.523	0.67	0.909	label-train
	LR	0.773	0.67	0.5472 -	0.811	0.5	0.922	0.265	0.922	0.811	0.863	0.891	label-test

			0.7921									
NaiveBayes	0.338	0.566	0.5040 - 0.6279	0.284	0.843	0.944	0.112	0.944	0.284	0.437	0.996	label-train
NaiveBayes	0.838	0.74	0.6255 - 0.8555	0.874	0.577	0.938	0.385	0.938	0.874	0.905	0.98	label-test
SVM	0.902	0.204	0.1452 - 0.2624	0.999	0	0.903	0	0.903	0.999	0.949	0.893	label-train
SVM	0.824	0.406	0.2579 - 0.5550	0.911	0.192	0.892	0.227	0.892	0.911	0.901	0.903	label-test
KNN	0.097	0.879	0.8560 - 0.9015	0	1	0	0.097	0	0	NaN	1	label-train
KNN	0.569	0.536	0.4242 - 0.6483	0.584	0.462	0.888	0.132	0.888	0.584	0.705	0.8	label-test
RandomForest	0.827	0.884	0.8481 - 0.9202	0.83	0.795	0.974	0.333	0.974	0.83	0.896	0.891	label-train
RandomForest	0.708	0.719	0.6164 - 0.8221	0.711	0.692	0.944	0.247	0.944	0.711	0.811	0.901	label-test
ExtraTrees	0.695	0.737	0.6807 - 0.7938	0.7	0.651	0.949	0.188	0.949	0.7	0.806	0.905	label-train
ExtraTrees	0.644	0.623	0.4935 - 0.7522	0.642	0.654	0.931	0.2	0.931	0.642	0.76	0.906	label-test
XGBoost	0.712	0.772	0.7146 - 0.8298	0.71	0.723	0.96	0.211	0.96	0.71	0.817	0.842	label-train
XGBoost	0.38	0.635	0.5275 - 0.7426	0.311	0.885	0.952	0.149	0.952	0.311	0.468	0.864	label-test
LightGBM	0.833	0.923	0.8952 -	0.828	0.88	0.985	0.353	0.985	0.828	0.899	0.877	label-train

				0.9514									
	LightGBM	0.463	0.589	0.4801 - 0.6988	0.416	0.808	0.94	0.159	0.94	0.416	0.577	0.929	label-test
	GradientBoosting	0.697	0.807	0.7552 - 0.8590	0.687	0.783	0.967	0.211	0.967	0.687	0.804	0.907	label-train
	GradientBoosting	0.773	0.604	0.4785 - 0.7290	0.816	0.462	0.917	0.255	0.917	0.816	0.864	0.89	label-test
	AdaBoost	0.55	0.756	0.7048 - 0.8062	0.519	0.843	0.969	0.158	0.969	0.519	0.676	0.565	label-train
	AdaBoost	0.648	0.647	0.5404 - 0.7531	0.658	0.577	0.919	0.187	0.919	0.658	0.767	0.553	label-test
	MLP	0.321	0.535	0.4698 - 0.6011	0.265	0.843	0.941	0.109	0.941	0.265	0.414	0.904	label-train
	MLP	0.815	0.465	0.3235 - 0.6061	0.895	0.231	0.895	0.231	0.895	0.895	0.895	0.885	label-test
Muscle_Pathological Classification													
	LR	0.282	0.565	0.5037 - 0.6268	0.208	0.902	0.947	0.12	0.947	0.208	0.341	0.918	label-train
	LR	0.681	0.64	0.5229 - 0.7571	0.692	0.556	0.945	0.141	0.945	0.692	0.799	0.881	label-test
	NaiveBayes	0.78	0.579	0.5168 - 0.6411	0.838	0.293	0.908	0.178	0.908	0.838	0.872	0.877	label-train
	NaiveBayes	0.597	0.646	0.5353 - 0.7571	0.586	0.722	0.959	0.137	0.959	0.586	0.727	0.9	label-test
	SVM	0.892	0.401	0.3410 -	0.999	0	0.893	0	0.893	0.999	0.943	0.731	label-train

			0.4614									
SVM	0.106	0.343	0.2125 - 0.4738	0.025	1	1	0.085	1	0.025	0.049	0.963	label-test
KNN	0.107	0.858	0.8346 - 0.8820	0	1	0	0.107	0	0	NaN	1	label-train
KNN	0.898	0.419	0.3106 - 0.5278	0.98	0	0.915	0	0.915	0.98	0.946	0.4	label-test
RandomForest	0.876	0.847	0.8062 - 0.8878	0.906	0.62	0.952	0.442	0.952	0.906	0.929	0.867	label-train
RandomForest	0.639	0.676	0.5622 - 0.7888	0.636	0.667	0.955	0.143	0.955	0.636	0.764	0.9	label-test
ExtraTrees	0.651	0.707	0.6511 - 0.7621	0.648	0.674	0.943	0.186	0.943	0.648	0.768	0.895	label-train
ExtraTrees	0.495	0.731	0.6438 - 0.8175	0.449	1	1	0.142	1	0.449	0.62	0.899	label-test
XGBoost	0.661	0.708	0.6527 - 0.7625	0.669	0.598	0.933	0.177	0.933	0.669	0.779	0.847	label-train
XGBoost	0.273	0.668	0.5692 - 0.7678	0.207	1	1	0.103	1	0.207	0.343	0.853	label-test
LightGBM	0.654	0.809	0.7677 - 0.8501	0.63	0.859	0.974	0.217	0.974	0.63	0.765	0.891	label-train
LightGBM	0.625	0.704	0.6041 - 0.8033	0.601	0.889	0.983	0.168	0.983	0.601	0.746	0.895	label-test
GradientBoosting	0.792	0.727	0.6744 - 0.7800	0.829	0.489	0.931	0.254	0.931	0.829	0.877	0.881	label-train
GradientBoosting	0.685	0.7	0.5955 -	0.687	0.667	0.958	0.162	0.958	0.687	0.8	0.89	label-test

				0.8052									
	AdaBoost	0.396	0.69	0.6403 - 0.7393	0.331	0.935	0.977	0.143	0.977	0.331	0.495	0.557	label-train
	AdaBoost	0.551	0.657	0.5517 - 0.7631	0.535	0.722	0.955	0.124	0.955	0.535	0.686	0.553	label-test
	MLP	0.589	0.52	0.4607 - 0.5792	0.603	0.478	0.906	0.126	0.906	0.603	0.724	0.877	label-train
	MLP	0.537	0.665	0.5558 - 0.7747	0.51	0.833	0.971	0.134	0.971	0.51	0.669	0.882	label-test
Esophageal_T Staging													
	SVM	0.623	0.859	0.8078-0.9109	0.931	0.614	0.064	0.997	0.064	0.931	0.120	0.028	train-label0
	SVM	0.755	0.851	0.8241-0.8788	0.841	0.735	0.438	0.949	0.438	0.841	0.576	0.160	train-label1
	SVM	0.710	0.755	0.7180-0.7920	0.691	0.716	0.448	0.874	0.448	0.691	0.544	0.270	train-label2
	SVM	0.740	0.776	0.7479-0.8045	0.722	0.759	0.759	0.723	0.759	0.722	0.740	0.567	train-label3
	SVM	0.806	0.735	0.5552-0.9151	0.538	0.809	0.034	0.993	0.034	0.538	0.065	0.014	train-label4
	SVM	0.527	0.537	1.0000-1.0000	0.000	0.537	0.000	0.967	0.000	0.000	NaN	0.028	test-label0
	SVM	0.836	0.847	0.7075-0.9863	0.750	0.860	0.600	0.925	0.600	0.750	0.667	0.272	test-label1
	SVM	0.473	0.595	0.4242-0.76	0.846	0.357	0.289	0.882	0.289	0.846	0.431	0.231	test-label2

			63									
SVM	0.673	0.648	0.4955-0.8008	0.857	0.481	0.632	0.765	0.632	0.857	0.727	0.474	test-label3
SVM	0.491	0.500	1.0000-1.0000	0.000	0.500	0.000	0.964	0.000	0.000	NaN	0.013	test-label4
KNN	0.976	0.957	0.9466-0.9679	0.172	0.999	0.833	0.977	0.833	0.172	0.286	0.200	train-label0
KNN	0.844	0.886	0.8658-0.9052	0.411	0.951	0.675	0.868	0.675	0.411	0.511	0.400	train-label1
KNN	0.780	0.784	0.7567-0.8104	0.275	0.949	0.643	0.797	0.643	0.275	0.385	0.400	train-label2
KNN	0.670	0.790	0.7638-0.8154	0.435	0.918	0.847	0.608	0.847	0.435	0.575	0.600	train-label3
KNN	0.988	0.985	0.9798-0.9902	0.000	1.000	0.000	0.988	0.000	0.000	NaN	0.200	train-label4
KNN	0.800	0.407	1.0000-1.0000	0.000	0.815	0.000	0.978	0.000	0.000	NaN	0.000	test-label0
KNN	0.745	0.747	0.6014-0.8928	0.333	0.860	0.400	0.822	0.400	0.333	0.364	0.400	test-label1
KNN	0.545	0.567	0.4153-0.7184	0.462	0.571	0.250	0.774	0.250	0.462	0.324	0.200	test-label2
KNN	0.509	0.637	0.4869-0.7869	0.179	0.852	0.556	0.500	0.556	0.179	0.270	0.600	test-label3
KNN	0.964	0.491	1.0000-1.0000	0.000	0.981	0.000	0.981	0.000	0.000	NaN	0.000	test-label4
RandomForest	0.998	1.000	1.0000-1.00	0.931	1.000	1.000	0.998	1.000	0.931	0.964	0.400	train-label0

			00									
RandomForest	0.992	1.000	0.9992-1.0000	0.971	0.998	0.990	0.993	0.990	0.971	0.980	0.400	train-label1
RandomForest	0.986	1.000	0.9996-1.0000	0.943	1.000	1.000	0.981	1.000	0.943	0.971	0.500	train-label2
RandomForest	0.979	0.999	0.9989-0.9999	0.961	0.998	0.998	0.960	0.998	0.961	0.979	0.500	train-label3
RandomForest	0.997	1.000	1.0000-1.0000	0.769	1.000	1.000	0.997	1.000	0.769	0.870	0.400	train-label4
RandomForest	0.727	0.370	1.0000-1.0000	0.000	0.741	0.000	0.976	0.000	0.000	NaN	0.000	test-label0
RandomForest	0.836	0.730	0.5430-0.9163	0.500	0.930	0.667	0.870	0.667	0.500	0.571	0.400	test-label1
RandomForest	0.782	0.689	0.5026-0.8747	0.154	0.976	0.667	0.788	0.667	0.154	0.250	0.400	test-label2
RandomForest	0.582	0.616	0.4642-0.7673	0.643	0.519	0.581	0.583	0.581	0.643	0.610	0.400	test-label3
RandomForest	0.964	0.907	1.0000-1.0000	0.000	0.981	0.000	0.981	0.000	0.000	NaN	0.100	test-label4
ExtraTrees	0.972	1.000	1.0000-1.0000	0.000	1.000	0.000	0.972	0.000	0.000	NaN	1.000	train-label0
ExtraTrees	0.802	1.000	1.0000-1.0000	0.000	1.000	0.000	0.802	0.000	0.000	NaN	1.000	train-label1
ExtraTrees	0.750	1.000	1.0000-1.0000	0.000	1.000	0.000	0.750	0.000	0.000	NaN	1.000	train-label2
ExtraTrees	0.488	1.000	1.0000-1.00	0.000	1.000	0.000	0.488	0.000	0.000	NaN	1.000	train-label3

			00									
ExtraTrees	0.988	1.000	1.0000-1.0000	0.000	1.000	0.000	0.988	0.000	0.000	NaN	1.000	train-label4
ExtraTrees	0.745	0.380	1.0000-1.0000	0.000	0.759	0.000	0.976	0.000	0.000	NaN	0.000	test-label0
ExtraTrees	0.782	0.744	0.5546-0.9338	0.500	0.860	0.500	0.860	0.500	0.500	0.500	0.300	test-label1
ExtraTrees	0.545	0.522	0.3638-0.6802	0.538	0.548	0.269	0.793	0.269	0.538	0.359	0.200	test-label2
ExtraTrees	0.564	0.661	0.5174-0.8053	0.357	0.778	0.625	0.538	0.625	0.357	0.455	0.600	test-label3
ExtraTrees	0.873	0.444	1.0000-1.0000	0.000	0.889	0.000	0.980	0.000	0.000	NaN	0.000	test-label4
XGBoost	0.988	0.996	0.9911-1.0000	0.931	0.989	0.711	0.998	0.711	0.931	0.806	0.119	train-label0
XGBoost	0.960	0.992	0.9885-0.9965	0.942	0.964	0.867	0.985	0.867	0.942	0.903	0.321	train-label1
XGBoost	0.950	0.994	0.9909-0.9968	0.973	0.943	0.850	0.991	0.850	0.973	0.907	0.247	train-label2
XGBoost	0.937	0.983	0.9773-0.9885	0.935	0.939	0.942	0.932	0.942	0.935	0.938	0.507	train-label3
XGBoost	0.868	0.980	0.9576-1.0000	0.923	0.868	0.081	0.999	0.081	0.923	0.148	0.049	train-label4
XGBoost	0.527	0.537	1.0000-1.0000	0.000	0.537	0.000	0.967	0.000	0.000	NaN	0.041	test-label0
XGBoost	0.873	0.866	0.7256-1.00	0.750	0.907	0.692	0.929	0.692	0.750	0.720	0.316	test-label1

			00								
XGBoost	0.618	0.711	0.5394-0.8818	0.769	0.571	0.357	0.889	0.357	0.769	0.488	0.191	test-label2
XGBoost	0.673	0.640	0.4865-0.7939	0.786	0.556	0.647	0.714	0.647	0.786	0.710	0.437	test-label3
XGBoost	0.036	0.037	1.0000-1.0000	0.000	0.037	0.000	0.667	0.000	0.000	NaN	0.024	test-label4
LightGBM	0.988	0.999	0.9980-1.0000	0.966	0.988	0.700	0.999	0.700	0.966	0.812	0.088	train-label0
LightGBM	0.904	0.969	0.9591-0.9783	0.889	0.907	0.702	0.971	0.702	0.889	0.785	0.269	train-label1
LightGBM	0.914	0.975	0.9669-0.9833	0.912	0.915	0.781	0.969	0.781	0.912	0.842	0.276	train-label2
LightGBM	0.879	0.951	0.9397-0.9628	0.890	0.867	0.875	0.882	0.875	0.890	0.883	0.497	train-label3
LightGBM	0.999	1.000	1.0000-1.0000	0.923	1.000	1.000	0.999	1.000	0.923	0.960	0.217	train-label4
LightGBM	0.145	0.148	1.0000-1.0000	0.000	0.148	0.000	0.889	0.000	0.000	NaN	0.013	test-label0
LightGBM	0.764	0.839	0.6864-0.9919	0.833	0.744	0.476	0.941	0.476	0.833	0.606	0.215	test-label1
LightGBM	0.491	0.650	0.4865-0.8139	0.846	0.381	0.297	0.889	0.297	0.846	0.440	0.175	test-label2
LightGBM	0.673	0.672	0.5262-0.8177	0.714	0.630	0.667	0.680	0.667	0.714	0.690	0.500	test-label3
LightGBM	0.036	0.037	1.0000-1.00	0.000	0.037	0.000	0.667	0.000	0.000	NaN	0.004	test-label4

			00										
NaiveBayes	0.590	0.717	0.6212-0.8132	0.724	0.586	0.048	0.987	0.048	0.724	0.089	0.025	train-label0
NaiveBayes	0.782	0.733	0.6922-0.7732	0.570	0.835	0.459	0.887	0.459	0.570	0.509	0.534	train-label1
NaiveBayes	0.518	0.585	0.5455-0.6249	0.679	0.464	0.297	0.812	0.297	0.679	0.413	0.194	train-label2
NaiveBayes	0.623	0.655	0.6221-0.6879	0.683	0.560	0.619	0.627	0.619	0.683	0.650	0.347	train-label3
NaiveBayes	0.772	0.766	0.6143-0.9173	0.692	0.773	0.037	0.995	0.037	0.692	0.070	0.007	train-label4
NaiveBayes	0.709	0.722	1.0000-1.0000	0.000	0.722	0.000	0.975	0.000	0.000	NaN	0.052	test-label0
NaiveBayes	0.818	0.845	0.7186-0.9713	0.750	0.837	0.562	0.923	0.562	0.750	0.643	0.524	test-label1
NaiveBayes	0.655	0.690	0.5136-0.8674	0.692	0.643	0.375	0.871	0.375	0.692	0.486	0.237	test-label2
NaiveBayes	0.636	0.663	0.5165-0.8089	0.571	0.704	0.667	0.613	0.667	0.571	0.615	0.428	test-label3
NaiveBayes	0.036	0.037	1.0000-1.0000	0.000	0.037	0.000	0.667	0.000	0.000	NaN	0.000	test-label4
AdaBoost	0.516	0.786	0.7164-0.8551	0.897	0.505	0.049	0.994	0.049	0.897	0.093	0.170	train-label0
AdaBoost	0.740	0.707	0.6678-0.7463	0.478	0.805	0.376	0.862	0.376	0.478	0.421	0.236	train-label1
AdaBoost	0.613	0.538	0.4974-0.57	0.389	0.688	0.294	0.771	0.294	0.389	0.335	0.243	train-label2

			84									
AdaBoost	0.578	0.577	0.5424-0.6113	0.709	0.440	0.571	0.591	0.571	0.709	0.632	0.248	train-label3
AdaBoost	0.972	0.790	0.6534-0.9269	0.385	0.980	0.192	0.992	0.192	0.385	0.256	0.167	train-label4
AdaBoost	0.364	0.361	1.0000-1.0000	0.000	0.370	0.000	0.952	0.000	0.000	NaN	0.170	test-label0
AdaBoost	0.691	0.654	0.4808-0.8274	0.500	0.744	0.353	0.842	0.353	0.500	0.414	0.236	test-label1
AdaBoost	0.418	0.541	0.3797-0.7027	0.923	0.262	0.279	0.917	0.279	0.923	0.429	0.224	test-label2
AdaBoost	0.582	0.608	0.4558-0.7598	0.500	0.667	0.609	0.562	0.609	0.500	0.549	0.250	test-label3
AdaBoost	0.182	0.185	1.0000-1.0000	0.000	0.185	0.000	0.909	0.000	0.000	NaN	0.066	test-label4
GradientBoosting	0.846	0.922	0.8612-0.9834	0.828	0.847	0.133	0.994	0.133	0.828	0.230	0.030	train-label0
GradientBoosting	0.689	0.866	0.8405-0.8924	0.874	0.643	0.376	0.954	0.376	0.874	0.526	0.169	train-label1
GradientBoosting	0.752	0.814	0.7842-0.8442	0.706	0.767	0.503	0.887	0.503	0.706	0.587	0.256	train-label2
GradientBoosting	0.728	0.807	0.7810-0.8324	0.679	0.779	0.763	0.698	0.763	0.679	0.719	0.532	train-label3
GradientBoosting	0.927	0.919	0.7951-1.0000	0.769	0.929	0.120	0.997	0.120	0.769	0.208	0.011	train-label4
GradientBoosting	0.582	0.593	1.0000-1.00	0.000	0.593	0.000	0.970	0.000	0.000	NaN	0.026	test-label0

			00									
GradientBoosting	0.727	0.831	0.6744-0.9884	0.833	0.698	0.435	0.937	0.435	0.833	0.571	0.200	test-label1
GradientBoosting	0.673	0.645	0.4877-0.8017	0.538	0.714	0.368	0.833	0.368	0.538	0.437	0.241	test-label2
GradientBoosting	0.673	0.677	0.5317-0.8228	0.607	0.741	0.708	0.645	0.708	0.607	0.654	0.529	test-label3
GradientBoosting	0.345	0.352	1.0000-1.0000	0.000	0.352	0.000	0.950	0.000	0.000	NaN	0.007	test-label4
MLP	0.576	0.768	0.6824-0.8531	0.793	0.570	0.050	0.990	0.050	0.793	0.094	0.028	train-label0
MLP	0.729	0.803	0.7705-0.8357	0.729	0.729	0.398	0.916	0.398	0.729	0.515	0.203	train-label1
MLP	0.664	0.699	0.6637-0.7347	0.637	0.673	0.394	0.848	0.394	0.637	0.487	0.255	train-label2
MLP	0.676	0.736	0.7063-0.7657	0.591	0.765	0.725	0.641	0.725	0.591	0.652	0.573	train-label3
MLP	0.621	0.728	0.5943-0.8621	0.769	0.619	0.025	0.995	0.025	0.769	0.048	0.016	train-label4
MLP	0.673	0.685	1.0000-1.0000	0.000	0.685	0.000	0.974	0.000	0.000	NaN	0.037	test-label0
MLP	0.836	0.851	0.7278-0.9738	0.833	0.837	0.588	0.947	0.588	0.833	0.690	0.292	test-label1
MLP	0.455	0.588	0.4110-0.7648	0.846	0.333	0.282	0.875	0.282	0.846	0.423	0.197	test-label2
MLP	0.709	0.706	0.5654-0.84	0.750	0.667	0.700	0.720	0.700	0.750	0.724	0.534	test-label3

				73									
	MLP	0.182	0.185	1.0000-1.0000	0.000	0.185	0.000	0.909	0.000	0.000	NaN	0.007	test-label4
Esophageal plus Stomach_T Staging													
	SVM	0.962	0.342	0.2263-0.4578	0.000	0.989	0.000	0.972	0.000	0.000	NaN	0.036	train-label0
	SVM	0.773	0.469	0.4248-0.5142	0.091	0.943	0.284	0.807	0.284	0.091	NaN	0.200	train-label1
	SVM	0.635	0.483	0.4418-0.5243	0.261	0.759	0.265	0.755	0.265	0.261	NaN	0.243	train-label2
	SVM	0.568	0.584	0.5494-0.6182	0.729	0.399	0.560	0.585	0.560	0.729	NaN	0.518	train-label3
	SVM	0.266	0.587	0.4338-0.7397	0.846	0.258	0.014	0.993	0.014	0.846	NaN	0.013	train-label4
	SVM	0.556	0.566	1.0000-1.0000	0.000	0.566	0.000	0.968	0.000	0.000	NaN	0.028	test-label0
	SVM	0.796	0.594	0.3929-0.7952	0.182	0.953	0.500	0.820	0.500	0.182	NaN	0.205	test-label1
	SVM	0.611	0.580	0.4045-0.7562	0.500	0.650	0.333	0.788	0.333	0.500	NaN	0.242	test-label2
	SVM	0.611	0.591	0.4349-0.7476	0.852	0.370	0.575	0.714	0.575	0.852	NaN	0.517	test-label3
	SVM	0.519	0.528	1.0000-1.0000	0.000	0.528	0.000	0.966	0.000	0.000	NaN	0.013	test-label4
	KNN	0.970	0.947	0.9364-0.95	0.069	0.996	0.333	0.974	0.333	0.069	NaN	0.200	train-label0

			79									
KNN	0.832	0.805	0.7783-0.8317	0.255	0.975	0.716	0.841	0.716	0.255	0.376	0.400	train-label1
KNN	0.780	0.800	0.7741-0.8264	0.287	0.944	0.630	0.799	0.630	0.287	0.395	0.400	train-label2
KNN	0.631	0.768	0.7410-0.7945	0.329	0.947	0.867	0.574	0.867	0.329	0.477	0.600	train-label3
KNN	0.988	0.978	0.9720-0.9844	0.000	1.000	0.000	0.988	0.000	0.000	NaN	0.200	train-label4
KNN	0.870	0.443	1.0000-1.0000	0.000	0.887	0.000	0.979	0.000	0.000	NaN	0.000	test-label0
KNN	0.796	0.585	0.3688-0.8003	0.091	0.977	0.500	0.808	0.500	0.091	0.154	0.400	test-label1
KNN	0.667	0.626	0.4754-0.7764	0.357	0.775	0.357	0.775	0.357	0.357	0.357	0.200	test-label2
KNN	0.611	0.680	0.5387-0.8221	0.444	0.778	0.667	0.583	0.667	0.444	0.533	0.600	test-label3
KNN	0.907	0.462	1.0000-1.0000	0.000	0.925	0.000	0.980	0.000	0.000	NaN	0.000	test-label4
RandomForest	0.999	1.000	1.0000-1.0000	0.966	1.000	1.000	0.999	1.000	0.966	0.982	0.300	train-label0
RandomForest	0.982	0.999	0.9978-0.9996	0.928	0.995	0.980	0.982	0.980	0.928	0.953	0.400	train-label1
RandomForest	0.989	0.999	0.9990-0.9999	0.973	0.995	0.984	0.991	0.984	0.973	0.979	0.400	train-label2
RandomForest	0.975	0.998	0.9974-0.99	0.963	0.988	0.988	0.962	0.988	0.963	0.975	0.500	train-label3

			94									
RandomForest	0.999	1.000	0.9996-1.0000	0.923	1.000	1.000	0.999	1.000	0.923	0.960	0.300	train-label4
RandomForest	0.778	0.396	1.0000-1.0000	0.000	0.792	0.000	0.977	0.000	0.000	NaN	0.000	test-label0
RandomForest	0.833	0.598	0.3731-0.8236	0.182	1.000	1.000	0.827	1.000	0.182	0.308	0.600	test-label1
RandomForest	0.537	0.638	0.4741-0.8009	0.643	0.500	0.310	0.800	0.310	0.643	0.419	0.100	test-label2
RandomForest	0.611	0.631	0.4797-0.7823	0.630	0.593	0.607	0.615	0.607	0.630	0.618	0.500	test-label3
RandomForest	0.944	0.481	1.0000-1.0000	0.000	0.962	0.000	0.981	0.000	0.000	NaN	0.000	test-label4
ExtraTrees	0.972	1.000	1.0000-1.0000	0.000	1.000	0.000	0.972	0.000	0.000	NaN	1.000	train-label0
ExtraTrees	0.801	1.000	1.0000-1.0000	0.000	1.000	0.000	0.801	0.000	0.000	NaN	1.000	train-label1
ExtraTrees	0.750	1.000	1.0000-1.0000	0.000	1.000	0.000	0.750	0.000	0.000	NaN	1.000	train-label2
ExtraTrees	0.489	1.000	1.0000-1.0000	0.000	1.000	0.000	0.489	0.000	0.000	NaN	1.000	train-label3
ExtraTrees	0.988	1.000	1.0000-1.0000	0.000	1.000	0.000	0.988	0.000	0.000	NaN	1.000	train-label4
ExtraTrees	0.852	0.434	1.0000-1.0000	0.000	0.868	0.000	0.979	0.000	0.000	NaN	0.000	test-label0
ExtraTrees	0.796	0.574	0.3709-0.77	0.000	1.000	0.000	0.796	0.000	0.000	NaN	1.000	test-label1

			70									
ExtraTrees	0.685	0.563	0.3856-0.7393	0.214	0.850	0.333	0.756	0.333	0.214	0.261	0.300	test-label2
ExtraTrees	0.611	0.700	0.5594-0.8398	0.407	0.815	0.687	0.579	0.687	0.407	0.512	0.700	test-label3
ExtraTrees	0.926	0.472	1.0000-1.0000	0.000	0.943	0.000	0.980	0.000	0.000	NaN	0.000	test-label4
XGBoost	0.972	0.966	0.9395-0.9918	0.793	0.977	0.500	0.994	0.500	0.793	0.613	0.121	train-label0
XGBoost	0.851	0.923	0.9042-0.9417	0.851	0.851	0.586	0.958	0.586	0.851	0.694	0.212	train-label1
XGBoost	0.831	0.913	0.8945-0.9314	0.831	0.831	0.620	0.937	0.620	0.831	0.710	0.260	train-label2
XGBoost	0.833	0.907	0.8902-0.9247	0.828	0.838	0.842	0.823	0.842	0.828	0.835	0.472	train-label3
XGBoost	0.824	0.904	0.8208-0.9872	0.769	0.825	0.052	0.996	0.052	0.769	0.098	0.046	train-label4
XGBoost	0.426	0.434	1.0000-1.0000	0.000	0.434	0.000	0.958	0.000	0.000	NaN	0.043	test-label0
XGBoost	0.574	0.698	0.5283-0.8671	0.727	0.535	0.286	0.885	0.286	0.727	0.410	0.175	test-label1
XGBoost	0.759	0.577	0.3873-0.7663	0.214	0.950	0.600	0.776	0.600	0.214	0.316	0.306	test-label2
XGBoost	0.648	0.684	0.5394-0.8296	0.667	0.630	0.643	0.654	0.643	0.667	0.655	0.470	test-label3
XGBoost	0.000	0.000	1.0000-1.00	0.000	0.000	0.000	0.000	0.000	0.000	NaN	0.022	test-label4

			00									
LightGBM	0.933	0.986	0.9785-0.9938	0.966	0.932	0.289	0.999	0.289	0.966	0.444	0.063	train-label0
LightGBM	0.839	0.904	0.8810-0.9268	0.793	0.851	0.569	0.943	0.569	0.793	0.663	0.234	train-label1
LightGBM	0.752	0.875	0.8516-0.8989	0.885	0.708	0.502	0.949	0.502	0.885	0.641	0.242	train-label2
LightGBM	0.781	0.861	0.8391-0.8831	0.779	0.783	0.790	0.772	0.790	0.779	0.785	0.504	train-label3
LightGBM	0.985	0.998	0.9955-1.0000	0.923	0.985	0.444	0.999	0.444	0.923	0.600	0.062	train-label4
LightGBM	0.204	0.208	1.0000-1.0000	0.000	0.208	0.000	0.917	0.000	0.000	NaN	0.012	test-label0
LightGBM	0.722	0.636	0.4415-0.8313	0.364	0.814	0.333	0.833	0.333	0.364	0.348	0.297	test-label1
LightGBM	0.500	0.614	0.4383-0.7903	0.786	0.400	0.314	0.842	0.314	0.786	0.449	0.196	test-label2
LightGBM	0.667	0.689	0.5434-0.8339	0.815	0.519	0.629	0.737	0.629	0.815	0.710	0.458	test-label3
LightGBM	0.870	0.887	1.0000-1.0000	0.000	0.887	0.000	0.979	0.000	0.000	NaN	0.017	test-label4
NaiveBayes	0.808	0.571	0.4496-0.6932	0.379	0.820	0.057	0.979	0.057	0.379	0.099	0.037	train-label0
NaiveBayes	0.587	0.564	0.5216-0.6064	0.519	0.604	0.245	0.835	0.245	0.519	0.333	0.187	train-label1
NaiveBayes	0.396	0.527	0.4876-0.56	0.820	0.255	0.268	0.810	0.268	0.820	0.404	0.259	train-label2

			66									
NaiveBayes	0.550	0.570	0.5352-0.6043	0.320	0.791	0.615	0.526	0.615	0.320	0.421	0.477	train-label3
NaiveBayes	0.614	0.635	0.4887-0.7821	0.615	0.614	0.020	0.992	0.020	0.615	0.038	0.036	train-label4
NaiveBayes	0.315	0.321	1.0000-1.0000	0.000	0.321	0.000	0.944	0.000	0.000	NaN	0.025	test-label0
NaiveBayes	0.815	0.560	0.3486-0.7719	0.182	0.977	0.667	0.824	0.667	0.182	0.286	0.293	test-label1
NaiveBayes	0.648	0.655	0.4830-0.8277	0.643	0.650	0.391	0.839	0.391	0.643	0.486	0.300	test-label2
NaiveBayes	0.630	0.653	0.5049-0.8010	0.741	0.519	0.606	0.667	0.606	0.741	0.667	0.438	test-label3
NaiveBayes	0.130	0.132	1.0000-1.0000	0.000	0.132	0.000	0.875	0.000	0.000	NaN	0.000	test-label4
AdaBoost	0.655	0.665	0.5691-0.7612	0.621	0.656	0.049	0.984	0.049	0.621	0.091	0.172	train-label0
AdaBoost	0.559	0.571	0.5284-0.6128	0.553	0.561	0.238	0.835	0.238	0.553	0.333	0.221	train-label1
AdaBoost	0.417	0.509	0.4699-0.5485	0.724	0.315	0.260	0.774	0.260	0.724	0.383	0.222	train-label2
AdaBoost	0.512	0.500	0.4647-0.5343	0.441	0.587	0.528	0.501	0.528	0.441	0.481	0.240	train-label3
AdaBoost	0.736	0.794	0.7138-0.8747	0.769	0.736	0.035	0.996	0.035	0.769	0.068	0.167	train-label4
AdaBoost	0.611	0.538	1.0000-1.00	0.000	0.623	0.000	0.971	0.000	0.000	NaN	0.167	test-label0

			00									
AdaBoost	0.407	0.506	0.3171-0.6956	0.636	0.349	0.200	0.789	0.200	0.636	0.304	0.216	test-label1
AdaBoost	0.759	0.728	0.5780-0.8774	0.500	0.850	0.538	0.829	0.538	0.500	0.519	0.229	test-label2
AdaBoost	0.519	0.480	0.3200-0.6402	0.667	0.370	0.514	0.526	0.514	0.667	0.581	0.240	test-label3
AdaBoost	0.981	0.972	1.0000-1.0000	0.000	1.000	0.000	0.981	0.000	0.000	NaN	0.213	test-label4
GradientBoosting	0.715	0.902	0.8542-0.9495	0.897	0.710	0.081	0.996	0.081	0.897	0.149	0.026	train-label0
GradientBoosting	0.617	0.752	0.7165-0.7865	0.788	0.574	0.315	0.916	0.315	0.788	0.450	0.203	train-label1
GradientBoosting	0.529	0.693	0.6575-0.7279	0.854	0.420	0.329	0.897	0.329	0.854	0.475	0.245	train-label2
GradientBoosting	0.638	0.701	0.6698-0.7319	0.785	0.483	0.614	0.682	0.614	0.785	0.689	0.499	train-label3
GradientBoosting	0.874	0.985	0.9644-1.0000	0.923	0.873	0.084	0.999	0.084	0.923	0.154	0.013	train-label4
GradientBoosting	0.667	0.679	1.0000-1.0000	0.000	0.679	0.000	0.973	0.000	0.000	NaN	0.026	test-label0
GradientBoosting	0.426	0.562	0.3830-0.7417	0.818	0.326	0.237	0.875	0.237	0.818	0.367	0.175	test-label1
GradientBoosting	0.722	0.563	0.3785-0.7483	0.286	0.875	0.444	0.778	0.444	0.286	0.348	0.263	test-label2
GradientBoosting	0.630	0.640	0.4891-0.79	0.444	0.815	0.706	0.595	0.706	0.444	0.545	0.528	test-label3

				07								
	GradientBoosting	0.037	0.038	1.0000-1.0000	0.000	0.038	0.000	0.667	0.000	0.000	NaN	0.006	test-label4
	MLP	0.794	0.651	0.5525-0.7502	0.414	0.805	0.057	0.980	0.057	0.414	0.100	0.030	train-label0
	MLP	0.572	0.576	0.5329-0.6187	0.582	0.569	0.251	0.846	0.251	0.582	0.351	0.201	train-label1
	MLP	0.459	0.545	0.5061-0.5844	0.728	0.369	0.277	0.803	0.277	0.728	0.402	0.248	train-label2
	MLP	0.554	0.576	0.5415-0.6105	0.641	0.462	0.555	0.551	0.555	0.641	0.595	0.502	train-label3
	MLP	0.789	0.454	0.2828-0.6246	0.231	0.796	0.014	0.988	0.014	0.231	0.026	0.020	train-label4
	MLP	0.444	0.453	1.0000-1.0000	0.000	0.453	0.000	0.960	0.000	0.000	NaN	0.027	test-label0
	MLP	0.833	0.641	0.4411-0.8400	0.273	0.977	0.750	0.840	0.750	0.273	0.400	0.249	test-label1
	MLP	0.537	0.666	0.5009-0.8313	0.786	0.450	0.333	0.857	0.333	0.786	0.468	0.248	test-label2
	MLP	0.648	0.652	0.5013-0.8019	0.815	0.481	0.611	0.722	0.611	0.815	0.698	0.496	test-label3
	MLP	0.037	0.038	1.0000-1.0000	0.000	0.038	0.000	0.667	0.000	0.000	NaN	0.003	test-label4
Esophageal_N Staging													
	SVM	0.699	0.711	0.6791-0.74	0.857	0.479	0.696	0.707	0.696	0.857	0.768	0.578	train-label0

			35									
SVM	0.662	0.755	0.7220-0.7886	0.710	0.647	0.389	0.876	0.389	0.710	0.503	0.245	train-label1
SVM	0.576	0.771	0.7297-0.8124	0.861	0.530	0.226	0.960	0.226	0.861	0.358	0.133	train-label2
SVM	0.039	0.143	0.0964-0.1896	0.976	0.000	0.039	0.000	0.039	0.976	0.075	0.021	train-label3
SVM	0.618	0.505	0.3348-0.6760	0.839	0.333	0.619	0.615	0.619	0.839	0.712	0.572	test-label0
SVM	0.709	0.411	0.2255-0.5968	0.071	0.927	0.250	0.745	0.250	0.071	0.111	0.254	test-label1
SVM	0.473	0.479	0.2465-0.7110	0.625	0.447	0.161	0.875	0.161	0.625	0.256	0.133	test-label2
SVM	0.691	0.802	0.5799-1.0000	0.500	0.698	0.059	0.974	0.059	0.500	0.105	0.041	test-label3
KNN	0.629	0.750	0.7219-0.7777	0.448	0.881	0.840	0.535	0.840	0.448	0.585	0.600	train-label0
KNN	0.788	0.795	0.7686-0.8214	0.258	0.956	0.650	0.803	0.650	0.258	0.369	0.400	train-label1
KNN	0.846	0.832	0.8081-0.8568	0.472	0.906	0.444	0.915	0.444	0.472	0.458	0.200	train-label2
KNN	0.955	0.926	0.9116-0.9410	0.190	0.987	0.381	0.967	0.381	0.190	0.254	0.200	train-label3
KNN	0.436	0.547	0.3950-0.6991	0.194	0.750	0.500	0.419	0.500	0.194	0.279	0.600	test-label0
KNN	0.218	0.274	0.1166-0.43	0.429	0.146	0.146	0.429	0.146	0.429	0.218	0.000	test-label1

			22									
KNN	0.382	0.355	0.1804-0.5297	0.375	0.383	0.094	0.783	0.094	0.375	0.150	0.000	test-label2
KNN	0.927	0.627	0.1517-1.0000	0.000	0.962	0.000	0.962	0.000	0.000	NaN	0.200	test-label3
RandomForest	0.958	0.999	0.9977-0.9994	0.928	1.000	1.000	0.909	1.000	0.928	NaN	0.600	train-label0
RandomForest	0.989	1.000	0.9991-1.0000	0.956	1.000	1.000	0.986	1.000	0.956	NaN	0.400	train-label1
RandomForest	0.991	0.999	0.9982-1.0000	0.951	0.998	0.986	0.992	0.986	0.951	0.968	0.400	train-label2
RandomForest	0.999	1.000	1.0000-1.0000	0.976	1.000	1.000	0.999	1.000	0.976	0.988	0.400	train-label3
RandomForest	0.655	0.767	0.6402-0.8948	0.484	0.875	0.833	0.568	0.833	0.484	0.612	0.600	test-label0
RandomForest	0.636	0.534	0.3545-0.7135	0.214	0.780	0.250	0.744	0.250	0.214	0.231	0.300	test-label1
RandomForest	0.855	0.653	0.4394-0.8665	0.000	1.000	0.000	0.855	0.000	0.000	NaN	0.500	test-label2
RandomForest	0.836	0.491	0.0578-0.9233	0.000	0.868	0.000	0.958	0.000	0.000	NaN	0.100	test-label3
ExtraTrees	0.418	1.000	1.0000-1.0000	0.000	1.000	0.000	0.418	0.000	0.000	NaN	1.000	train-label0
ExtraTrees	0.759	1.000	1.0000-1.0000	0.000	1.000	0.000	0.759	0.000	0.000	NaN	1.000	train-label1
ExtraTrees	0.862	1.000	1.0000-1.00	0.000	1.000	0.000	0.862	0.000	0.000	NaN	1.000	train-label2

			00									
ExtraTrees	0.960	1.000	1.0000-1.0000	0.000	1.000	0.000	0.960	0.000	0.000	NaN	1.000	train-label3
ExtraTrees	0.655	0.632	0.4763-0.7885	0.806	0.458	0.658	0.647	0.658	0.806	0.725	0.400	test-label0
ExtraTrees	0.745	0.451	0.2616-0.6408	0.000	1.000	0.000	0.745	0.000	0.000	NaN	0.500	test-label1
ExtraTrees	0.891	0.563	0.2920-0.8330	0.250	1.000	1.000	0.887	1.000	0.250	0.400	0.600	test-label2
ExtraTrees	0.927	0.580	0.1038-1.0000	0.000	0.962	0.000	0.962	0.000	0.000	NaN	0.100	test-label3
XGBoost	0.892	0.961	0.9505-0.9710	0.900	0.881	0.913	0.864	0.913	0.900	0.907	0.535	train-label0
XGBoost	0.883	0.967	0.9563-0.9774	0.929	0.868	0.690	0.975	0.690	0.929	0.792	0.255	train-label1
XGBoost	0.949	0.976	0.9643-0.9871	0.868	0.962	0.786	0.979	0.786	0.868	0.825	0.215	train-label2
XGBoost	0.947	0.981	0.9690-0.9932	0.881	0.949	0.420	0.995	0.420	0.881	0.569	0.116	train-label3
XGBoost	0.636	0.716	0.5817-0.8511	0.452	0.875	0.824	0.553	0.824	0.452	0.583	0.597	test-label0
XGBoost	0.418	0.467	0.2973-0.6365	0.714	0.317	0.263	0.765	0.263	0.714	0.385	0.202	test-label1
XGBoost	0.545	0.681	0.4768-0.8849	0.750	0.511	0.207	0.923	0.207	0.750	0.324	0.120	test-label2
XGBoost	0.218	0.349	0.0516-0.64	0.500	0.208	0.023	0.917	0.023	0.500	0.044	0.043	test-label3

			65									
LightGBM	0.858	0.936	0.9217-0.9500	0.851	0.868	0.899	0.807	0.899	0.851	0.874	0.566	train-label0
LightGBM	0.896	0.954	0.9411-0.9678	0.869	0.904	0.742	0.956	0.742	0.869	0.801	0.283	train-label1
LightGBM	0.883	0.962	0.9502-0.9744	0.896	0.882	0.547	0.982	0.547	0.896	0.679	0.177	train-label2
LightGBM	0.937	0.994	0.9891-0.9990	0.976	0.935	0.387	0.999	0.387	0.976	0.554	0.074	train-label3
LightGBM	0.709	0.698	0.5495-0.8457	0.710	0.708	0.759	0.654	0.759	0.710	0.733	0.554	test-label0
LightGBM	0.600	0.535	0.3585-0.7112	0.429	0.659	0.300	0.771	0.300	0.429	0.353	0.268	test-label1
LightGBM	0.527	0.676	0.4968-0.8543	0.750	0.489	0.200	0.920	0.200	0.750	0.316	0.128	test-label2
LightGBM	0.218	0.406	0.0041-0.8072	0.500	0.208	0.023	0.917	0.023	0.500	0.044	0.020	test-label3
NaiveBayes	0.527	0.573	0.5377-0.6075	0.386	0.724	0.660	0.459	0.660	0.386	0.487	0.630	train-label0
NaiveBayes	0.340	0.525	0.4848-0.5650	0.905	0.161	0.255	0.842	0.255	0.905	0.398	0.143	train-label1
NaiveBayes	0.682	0.632	0.5822-0.6826	0.493	0.712	0.215	0.898	0.215	0.493	0.299	0.116	train-label2
NaiveBayes	0.534	0.702	0.6369-0.7673	0.810	0.522	0.066	0.985	0.066	0.810	0.122	0.037	train-label3
NaiveBayes	0.727	0.767	0.6373-0.89	0.742	0.708	0.767	0.680	0.767	0.742	0.754	0.579	test-label0

			77									
NaiveBayes	0.455	0.495	0.3258-0.6637	0.643	0.390	0.265	0.762	0.265	0.643	0.375	0.204	test-label1
NaiveBayes	0.564	0.699	0.5140-0.8850	0.625	0.553	0.192	0.897	0.192	0.625	0.294	0.078	test-label2
NaiveBayes	0.564	0.292	0.0000-0.8695	0.000	0.585	0.000	0.939	0.000	0.000	NaN	0.037	test-label3
AdaBoost	0.528	0.571	0.5363-0.6053	0.365	0.756	0.675	0.461	0.675	0.365	0.473	0.297	train-label0
AdaBoost	0.551	0.528	0.4894-0.5670	0.512	0.564	0.271	0.785	0.271	0.512	0.354	0.267	train-label1
AdaBoost	0.324	0.574	0.5272-0.6208	0.889	0.234	0.156	0.930	0.156	0.889	0.266	0.241	train-label2
AdaBoost	0.663	0.774	0.7284-0.8196	0.762	0.659	0.085	0.985	0.085	0.762	0.153	0.217	train-label3
AdaBoost	0.618	0.600	0.4460-0.7543	0.548	0.708	0.708	0.548	0.708	0.548	0.618	0.297	test-label0
AdaBoost	0.218	0.275	0.1021-0.4484	0.857	0.000	0.226	0.000	0.226	0.857	0.358	0.238	test-label1
AdaBoost	0.800	0.682	0.4696-0.8948	0.375	0.872	0.333	0.891	0.333	0.375	0.353	0.262	test-label2
AdaBoost	0.455	0.500	0.3311-0.6689	0.500	0.453	0.033	0.960	0.033	0.500	0.062	0.193	test-label3
GradientBoosting	0.683	0.751	0.7213-0.7800	0.681	0.685	0.750	0.607	0.750	0.681	0.714	0.574	train-label0
GradientBoosting	0.678	0.749	0.7158-0.78	0.679	0.678	0.400	0.869	0.400	0.679	0.504	0.254	train-label1

			32								
GradientBoosting	0.725	0.767	0.7232-0.8107	0.653	0.736	0.283	0.930	0.283	0.653	0.395	0.142	train-label2
GradientBoosting	0.686	0.859	0.8049-0.9124	0.857	0.679	0.100	0.991	0.100	0.857	0.180	0.046	train-label3
GradientBoosting	0.673	0.704	0.5648-0.8425	0.645	0.708	0.741	0.607	0.741	0.645	0.690	0.570	test-label0
GradientBoosting	0.436	0.511	0.3495-0.6732	0.786	0.317	0.282	0.812	0.282	0.786	0.415	0.222	test-label1
GradientBoosting	0.618	0.633	0.4140-0.8519	0.625	0.617	0.217	0.906	0.217	0.625	0.323	0.141	test-label2
GradientBoosting	0.527	0.547	0.4094-0.6849	0.500	0.528	0.038	0.966	0.038	0.500	0.071	0.043	test-label3
MLP	0.595	0.625	0.5913-0.6597	0.558	0.646	0.687	0.513	0.687	0.558	0.616	0.576	train-label0
MLP	0.650	0.611	0.5700-0.6522	0.488	0.702	0.342	0.812	0.342	0.488	0.402	0.258	train-label1
MLP	0.622	0.679	0.6317-0.7272	0.694	0.610	0.221	0.926	0.221	0.694	0.336	0.141	train-label2
MLP	0.534	0.713	0.6442-0.7825	0.810	0.522	0.066	0.985	0.066	0.810	0.122	0.041	train-label3
MLP	0.691	0.735	0.6015-0.8689	0.581	0.833	0.818	0.606	0.818	0.581	0.679	0.605	test-label0
MLP	0.673	0.613	0.4453-0.7811	0.500	0.732	0.389	0.811	0.389	0.500	0.437	0.255	test-label1
MLP	0.655	0.625	0.4236-0.82	0.375	0.702	0.176	0.868	0.176	0.375	0.240	0.158	test-label2

				64								
	MLP	0.436	0.443	0.3084-0.5784	0.500	0.434	0.032	0.958	0.032	0.500	0.061	0.036	test-label3
Esophageal plus Stomach_N Staging												
	SVM	0.580	0.383	0.3488-0.4180	0.997	0.002	0.581	0.333	0.581	0.997	0.734	0.491	train-label0
	SVM	0.241	0.390	0.3510-0.4282	0.996	0.000	0.241	0.000	0.241	0.996	0.389	0.027	train-label1
	SVM	0.137	0.374	0.3256-0.4230	0.993	0.001	0.136	0.500	0.136	0.993	0.239	0.067	train-label2
	SVM	0.039	0.239	0.1624-0.3156	0.976	0.000	0.039	0.000	0.039	0.976	0.076	0.014	train-label3
	SVM	0.600	0.446	0.2735-0.6178	0.875	0.217	0.609	0.556	0.609	0.875	0.718	0.570	test-label0
	SVM	0.600	0.416	0.2198-0.6117	0.231	0.714	0.200	0.750	0.200	0.231	0.214	0.249	test-label1
	SVM	0.764	0.559	0.3135-0.8035	0.250	0.851	0.222	0.870	0.222	0.250	0.235	0.139	test-label2
	SVM	0.527	0.736	0.3209-1.0000	0.500	0.528	0.038	0.966	0.038	0.500	0.071	0.040	test-label3
	KNN	0.631	0.767	0.7391-0.7939	0.451	0.879	0.838	0.536	0.838	0.451	0.587	0.600	train-label0
	KNN	0.775	0.772	0.7444-0.8000	0.237	0.947	0.588	0.795	0.588	0.237	0.338	0.400	train-label1
	KNN	0.846	0.831	0.8075-0.85	0.413	0.915	0.434	0.908	0.434	0.413	0.423	0.200	train-label2

			42									
KNN	0.957	0.929	0.9147-0.9432	0.190	0.989	0.421	0.967	0.421	0.190	0.262	0.200	train-label3
KNN	0.582	0.730	0.5926-0.8666	0.437	0.783	0.737	0.500	0.737	0.437	0.549	0.600	test-label0
KNN	0.800	0.712	0.5337-0.8893	0.231	0.976	0.750	0.804	0.750	0.231	0.353	0.400	test-label1
KNN	0.836	0.588	0.3601-0.8154	0.000	0.979	0.000	0.852	0.000	0.000	NaN	0.400	test-label2
KNN	0.836	0.434	0.3880-0.4800	0.000	0.868	0.000	0.958	0.000	0.000	NaN	0.000	test-label3
RandomForest	0.955	0.999	0.9976-0.9995	0.924	0.998	0.998	0.905	0.998	0.924	0.960	0.600	train-label0
RandomForest	0.987	0.999	0.9981-0.9998	0.972	0.991	0.972	0.991	0.972	0.972	0.972	0.400	train-label1
RandomForest	0.991	1.000	0.9995-1.0000	0.937	1.000	1.000	0.990	1.000	0.937	0.968	0.400	train-label2
RandomForest	0.994	1.000	0.9998-1.0000	0.857	1.000	1.000	0.994	1.000	0.857	0.923	0.400	train-label3
RandomForest	0.491	0.617	0.4671-0.7666	0.187	0.913	0.750	0.447	0.750	0.187	0.300	0.700	test-label0
RandomForest	0.764	0.615	0.4187-0.8121	0.231	0.929	0.500	0.796	0.500	0.231	0.316	0.500	test-label1
RandomForest	0.836	0.573	0.3324-0.8139	0.000	0.979	0.000	0.852	0.000	0.000	NaN	0.400	test-label2
RandomForest	0.655	0.340	0.2762-0.40	0.000	0.679	0.000	0.947	0.000	0.000	NaN	0.000	test-label3

			31									
ExtraTrees	0.419	1.000	1.0000-1.0000	0.000	1.000	0.000	0.419	0.000	0.000	NaN	1.000	train-label0
ExtraTrees	0.758	1.000	1.0000-1.0000	0.000	1.000	0.000	0.758	0.000	0.000	NaN	1.000	train-label1
ExtraTrees	0.863	1.000	1.0000-1.0000	0.000	1.000	0.000	0.863	0.000	0.000	NaN	1.000	train-label2
ExtraTrees	0.960	1.000	1.0000-1.0000	0.000	1.000	0.000	0.960	0.000	0.000	NaN	1.000	train-label3
ExtraTrees	0.655	0.686	0.5407-0.8316	0.844	0.391	0.659	0.643	0.659	0.844	0.740	0.300	test-label0
ExtraTrees	0.745	0.703	0.5089-0.8976	0.462	0.833	0.462	0.833	0.462	0.462	0.462	0.400	test-label1
ExtraTrees	0.727	0.524	0.2977-0.7502	0.000	0.851	0.000	0.833	0.000	0.000	NaN	0.300	test-label2
ExtraTrees	0.655	0.340	0.2762-0.4031	0.000	0.679	0.000	0.947	0.000	0.000	NaN	0.000	test-label3
XGBoost	0.826	0.925	0.9094-0.9397	0.789	0.877	0.899	0.750	0.899	0.789	0.840	0.553	train-label0
XGBoost	0.819	0.937	0.9221-0.9514	0.933	0.783	0.578	0.973	0.578	0.933	0.714	0.249	train-label1
XGBoost	0.928	0.972	0.9582-0.9858	0.930	0.928	0.672	0.988	0.672	0.930	0.780	0.177	train-label2
XGBoost	0.895	0.960	0.9249-0.9954	0.905	0.894	0.264	0.996	0.264	0.905	0.409	0.087	train-label3
XGBoost	0.655	0.632	0.4767-0.78	0.781	0.478	0.676	0.611	0.676	0.781	0.725	0.455	test-label0

			69									
XGBoost	0.818	0.721	0.5358-0.9056	0.538	0.905	0.636	0.864	0.636	0.538	0.583	0.326	test-label1
XGBoost	0.655	0.412	0.1555-0.6690	0.250	0.723	0.133	0.850	0.133	0.250	0.174	0.160	test-label2
XGBoost	0.091	0.165	0.0000-0.3752	0.500	0.075	0.020	0.800	0.020	0.500	0.038	0.038	test-label3
LightGBM	0.809	0.881	0.8600-0.9013	0.811	0.806	0.853	0.754	0.853	0.811	0.831	0.576	train-label0
LightGBM	0.833	0.906	0.8848-0.9269	0.822	0.837	0.617	0.936	0.617	0.822	0.705	0.269	train-label1
LightGBM	0.834	0.920	0.8958-0.9444	0.846	0.833	0.445	0.972	0.445	0.846	0.583	0.163	train-label2
LightGBM	0.880	0.981	0.9709-0.9914	0.976	0.876	0.248	0.999	0.248	0.976	0.396	0.064	train-label3
LightGBM	0.727	0.675	0.5179-0.8327	0.812	0.609	0.743	0.700	0.743	0.812	0.776	0.512	test-label0
LightGBM	0.836	0.780	0.6282-0.9322	0.615	0.905	0.667	0.884	0.667	0.615	0.640	0.333	test-label1
LightGBM	0.582	0.473	0.2292-0.7176	0.375	0.617	0.143	0.853	0.143	0.375	0.207	0.146	test-label2
LightGBM	0.164	0.236	0.0412-0.4305	0.500	0.151	0.022	0.889	0.022	0.500	0.042	0.018	test-label3
NaiveBayes	0.522	0.552	0.5170-0.5874	0.353	0.758	0.669	0.458	0.669	0.353	0.462	0.487	train-label0
NaiveBayes	0.586	0.564	0.5227-0.60	0.545	0.598	0.303	0.805	0.303	0.545	0.389	0.331	train-label1

			45									
NaiveBayes	0.299	0.511	0.4621-0.5607	0.839	0.213	0.145	0.893	0.145	0.839	0.247	0.129	train-label2
NaiveBayes	0.799	0.657	0.5652-0.7498	0.476	0.813	0.096	0.974	0.096	0.476	0.160	0.107	train-label3
NaiveBayes	0.618	0.549	0.3857-0.7121	0.625	0.609	0.690	0.538	0.690	0.625	0.656	0.443	test-label0
NaiveBayes	0.327	0.480	0.2957-0.6640	0.923	0.143	0.250	0.857	0.250	0.923	0.393	0.095	test-label1
NaiveBayes	0.618	0.715	0.5644-0.8665	0.750	0.596	0.240	0.933	0.240	0.750	0.364	0.144	test-label2
NaiveBayes	0.527	0.274	0.0000-0.8141	0.000	0.547	0.000	0.935	0.000	0.000	NaN	0.074	test-label3
AdaBoost	0.506	0.564	0.5292-0.5992	0.395	0.660	0.617	0.441	0.617	0.395	0.482	0.284	train-label0
AdaBoost	0.574	0.541	0.5008-0.5815	0.518	0.592	0.289	0.794	0.289	0.518	0.371	0.264	train-label1
AdaBoost	0.458	0.528	0.4790-0.5771	0.601	0.436	0.145	0.873	0.145	0.601	0.233	0.247	train-label2
AdaBoost	0.650	0.775	0.7218-0.8276	0.738	0.646	0.080	0.983	0.080	0.738	0.145	0.217	train-label3
AdaBoost	0.582	0.541	0.3833-0.6996	0.812	0.261	0.605	0.500	0.605	0.812	0.693	0.275	test-label0
AdaBoost	0.582	0.391	0.1957-0.5863	0.231	0.690	0.187	0.744	0.187	0.231	0.207	0.269	test-label1
AdaBoost	0.855	0.448	0.1969-0.69	0.000	1.000	0.000	0.855	0.000	0.000	NaN	0.307	test-label2

			93									
AdaBoost	0.109	0.108	0.0000-0.2434	0.500	0.094	0.020	0.833	0.020	0.500	0.039	0.008	test-label3
GradientBoosting	0.704	0.750	0.7199-0.7801	0.773	0.610	0.733	0.659	0.733	0.773	0.752	0.561	train-label0
GradientBoosting	0.737	0.768	0.7356-0.8005	0.613	0.777	0.467	0.863	0.467	0.613	0.530	0.262	train-label1
GradientBoosting	0.702	0.767	0.7256-0.8090	0.664	0.708	0.265	0.930	0.265	0.664	0.379	0.141	train-label2
GradientBoosting	0.884	0.841	0.7766-0.9061	0.619	0.895	0.198	0.982	0.198	0.619	0.301	0.055	train-label3
GradientBoosting	0.600	0.592	0.4377-0.7471	0.469	0.783	0.750	0.514	0.750	0.469	0.577	0.578	test-label0
GradientBoosting	0.582	0.611	0.4276-0.7940	0.615	0.571	0.308	0.828	0.308	0.615	0.410	0.253	test-label1
GradientBoosting	0.582	0.316	0.0968-0.5361	0.250	0.638	0.105	0.833	0.105	0.250	0.148	0.142	test-label2
GradientBoosting	0.218	0.250	0.1010-0.3990	0.500	0.208	0.023	0.917	0.023	0.500	0.044	0.028	test-label3
MLP	0.565	0.579	0.5438-0.6140	0.509	0.642	0.663	0.485	0.663	0.509	0.576	0.571	train-label0
MLP	0.505	0.591	0.5504-0.6316	0.715	0.438	0.289	0.828	0.289	0.715	0.412	0.236	train-label1
MLP	0.442	0.541	0.4922-0.5891	0.713	0.399	0.158	0.898	0.158	0.713	0.259	0.135	train-label2
MLP	0.802	0.657	0.5628-0.75	0.476	0.816	0.098	0.974	0.098	0.476	0.162	0.054	train-label3

				18									
	MLP	0.527	0.507	0.3458-0.6678	0.375	0.739	0.667	0.459	0.667	0.375	0.480	0.581	test-label0
	MLP	0.382	0.511	0.3373-0.6847	0.846	0.238	0.256	0.833	0.256	0.846	0.393	0.222	test-label1
	MLP	0.345	0.598	0.4069-0.7899	0.875	0.255	0.167	0.923	0.167	0.875	0.280	0.128	test-label2
	MLP	0.600	0.311	0.0000-0.9250	0.000	0.623	0.000	0.943	0.000	0.000	NaN	0.051	test-label3
Muscle_N Staging													
	SVM	0.612	0.461	0.4240-0.4977	0.944	0.150	0.607	0.660	0.607	0.944	0.739	0.572	train-label0
	SVM	0.240	0.303	0.2639-0.3412	0.996	0.000	0.241	0.000	0.241	0.996	0.388	0.200	train-label1
	SVM	0.689	0.708	0.6627-0.7539	0.590	0.705	0.241	0.915	0.241	0.590	0.343	0.139	train-label2
	SVM	0.795	0.810	0.7327-0.8882	0.738	0.797	0.132	0.986	0.132	0.738	0.224	0.043	train-label3
	SVM	0.593	0.457	0.2945-0.6199	0.903	0.174	0.596	0.571	0.596	0.903	0.718	0.571	test-label0
	SVM	0.704	0.597	0.3891-0.8041	0.462	0.780	0.400	0.821	0.400	0.462	0.429	0.247	test-label1
	SVM	0.185	0.367	0.1567-0.5770	0.875	0.065	0.140	0.750	0.140	0.875	0.241	0.132	test-label2
	SVM	0.204	0.192	0.0841-0.30	0.500	0.192	0.023	0.909	0.023	0.500	0.044	0.037	test-label3

			05									
KNN	0.631	0.757	0.7296-0.7847	0.450	0.882	0.840	0.536	0.840	0.450	0.586	0.600	train-label0
KNN	0.801	0.814	0.7891-0.8399	0.296	0.961	0.708	0.811	0.708	0.296	0.418	0.400	train-label1
KNN	0.842	0.841	0.8174-0.8649	0.514	0.894	0.435	0.920	0.435	0.514	0.471	0.200	train-label2
KNN	0.962	0.942	0.9292-0.9547	0.214	0.993	0.562	0.968	0.562	0.214	0.310	0.200	train-label3
KNN	0.556	0.562	0.4121-0.7113	0.742	0.304	0.590	0.467	0.590	0.742	0.657	0.400	test-label0
KNN	0.611	0.558	0.3886-0.7277	0.308	0.707	0.250	0.763	0.250	0.308	0.276	0.200	test-label1
KNN	0.870	0.605	0.3641-0.8451	0.125	1.000	1.000	0.868	1.000	0.125	0.222	0.400	test-label2
KNN	0.833	0.433	0.3859-0.4795	0.000	0.865	0.000	0.957	0.000	0.000	NaN	0.000	test-label3
RandomForest	0.959	0.999	0.9984-0.9999	0.931	0.998	0.998	0.912	0.998	0.931	0.963	0.600	train-label0
RandomForest	0.989	0.998	0.9971-0.9999	0.964	0.996	0.988	0.989	0.988	0.964	0.976	0.400	train-label1
RandomForest	0.991	0.998	0.9946-1.0000	0.944	0.999	0.993	0.991	0.993	0.944	0.968	0.400	train-label2
RandomForest	0.997	1.000	1.0000-1.0000	0.929	1.000	1.000	0.997	1.000	0.929	0.963	0.400	train-label3
RandomForest	0.574	0.448	0.2866-0.60	0.968	0.043	0.577	0.500	0.577	0.968	0.723	0.100	test-label0

			96									
RandomForest	0.611	0.402	0.2114-0.5934	0.077	0.780	0.100	0.727	0.100	0.077	0.087	0.400	test-label1
RandomForest	0.815	0.558	0.3340-0.7828	0.000	0.957	0.000	0.846	0.000	0.000	NaN	0.300	test-label2
RandomForest	0.556	0.288	0.2207-0.3563	0.000	0.577	0.000	0.937	0.000	0.000	NaN	0.000	test-label3
ExtraTrees	0.419	1.000	1.0000-1.0000	0.000	1.000	0.000	0.419	0.000	0.000	NaN	1.000	train-label0
ExtraTrees	0.759	1.000	1.0000-1.0000	0.000	1.000	0.000	0.759	0.000	0.000	NaN	1.000	train-label1
ExtraTrees	0.863	1.000	1.0000-1.0000	0.000	1.000	0.000	0.863	0.000	0.000	NaN	1.000	train-label2
ExtraTrees	0.960	1.000	1.0000-1.0000	0.000	1.000	0.000	0.960	0.000	0.000	NaN	1.000	train-label3
ExtraTrees	0.556	0.582	0.4293-0.7348	0.710	0.348	0.595	0.471	0.595	0.710	0.647	0.400	test-label0
ExtraTrees	0.556	0.536	0.3511-0.7202	0.462	0.585	0.261	0.774	0.261	0.462	0.333	0.200	test-label1
ExtraTrees	0.648	0.624	0.4242-0.8231	0.500	0.674	0.211	0.886	0.211	0.500	0.296	0.100	test-label2
ExtraTrees	0.667	0.346	0.2828-0.4095	0.000	0.692	0.000	0.947	0.000	0.000	NaN	0.000	test-label3
XGBoost	0.854	0.932	0.9169-0.9462	0.880	0.818	0.870	0.831	0.870	0.880	0.875	0.540	train-label0
XGBoost	0.876	0.943	0.9281-0.95	0.830	0.891	0.707	0.943	0.707	0.830	0.764	0.267	train-label1

			70									
XGBoost	0.876	0.964	0.9518-0.9770	0.917	0.869	0.528	0.985	0.528	0.917	0.670	0.169	train-label2
XGBoost	0.909	0.954	0.9268-0.9805	0.810	0.914	0.281	0.991	0.281	0.810	0.417	0.093	train-label3
XGBoost	0.630	0.568	0.4078-0.7283	0.710	0.522	0.667	0.571	0.667	0.710	0.687	0.489	test-label0
XGBoost	0.685	0.510	0.3078-0.7128	0.308	0.805	0.333	0.786	0.333	0.308	0.320	0.301	test-label1
XGBoost	0.630	0.625	0.4130-0.8370	0.500	0.652	0.200	0.882	0.200	0.500	0.286	0.155	test-label2
XGBoost	0.870	0.519	0.0000-1.0000	0.000	0.904	0.000	0.959	0.000	0.000	NaN	0.098	test-label3
LightGBM	0.834	0.912	0.8951-0.9295	0.824	0.847	0.882	0.777	0.882	0.824	0.852	0.570	train-label0
LightGBM	0.849	0.928	0.9087-0.9465	0.885	0.838	0.635	0.958	0.635	0.885	0.739	0.269	train-label1
LightGBM	0.921	0.964	0.9509-0.9779	0.875	0.928	0.660	0.979	0.660	0.875	0.752	0.195	train-label2
LightGBM	0.963	0.990	0.9825-0.9974	0.929	0.964	0.520	0.997	0.520	0.929	0.667	0.094	train-label3
LightGBM	0.537	0.546	0.3878-0.7033	0.355	0.783	0.687	0.474	0.687	0.355	0.468	0.621	test-label0
LightGBM	0.352	0.439	0.2505-0.6276	0.769	0.220	0.238	0.750	0.238	0.769	0.364	0.167	test-label1
LightGBM	0.778	0.709	0.5091-0.90	0.625	0.804	0.357	0.925	0.357	0.625	0.455	0.184	test-label2

			94									
LightGBM	0.259	0.269	0.1447-0.3938	0.500	0.250	0.025	0.929	0.025	0.500	0.048	0.020	test-label3
NaiveBayes	0.575	0.561	0.5257-0.5959	0.722	0.371	0.615	0.491	0.615	0.722	0.664	0.524	train-label0
NaiveBayes	0.561	0.550	0.5100-0.5904	0.518	0.575	0.279	0.789	0.279	0.518	0.363	0.257	train-label1
NaiveBayes	0.563	0.592	0.5419-0.6422	0.604	0.556	0.178	0.898	0.178	0.604	0.275	0.130	train-label2
NaiveBayes	0.554	0.639	0.5548-0.7241	0.690	0.549	0.060	0.977	0.060	0.690	0.110	0.036	train-label3
NaiveBayes	0.667	0.663	0.5098-0.8170	0.613	0.739	0.760	0.586	0.760	0.613	0.679	0.566	test-label0
NaiveBayes	0.648	0.443	0.2500-0.6355	0.231	0.780	0.250	0.762	0.250	0.231	0.240	0.284	test-label1
NaiveBayes	0.704	0.747	0.5606-0.9340	0.625	0.717	0.278	0.917	0.278	0.625	0.385	0.162	test-label2
NaiveBayes	0.407	0.462	0.2903-0.6328	0.500	0.404	0.031	0.955	0.031	0.500	0.059	0.029	test-label3
AdaBoost	0.493	0.540	0.5064-0.5742	0.274	0.797	0.652	0.442	0.652	0.274	0.386	0.289	train-label0
AdaBoost	0.355	0.540	0.5016-0.5778	0.885	0.186	0.257	0.836	0.257	0.885	0.399	0.257	train-label1
AdaBoost	0.778	0.545	0.4946-0.5952	0.187	0.872	0.189	0.871	0.189	0.187	0.188	0.254	train-label2
AdaBoost	0.717	0.740	0.6739-0.80	0.667	0.719	0.090	0.981	0.090	0.667	0.159	0.215	train-label3

			61									
AdaBoost	0.500	0.409	0.2557-0.5620	0.290	0.783	0.643	0.450	0.643	0.290	0.400	0.287	test-label0
AdaBoost	0.630	0.622	0.4492-0.7947	0.385	0.707	0.294	0.784	0.294	0.385	0.333	0.261	test-label1
AdaBoost	0.630	0.698	0.5224-0.8743	0.500	0.652	0.200	0.882	0.200	0.500	0.286	0.246	test-label2
AdaBoost	0.667	0.538	0.4248-0.6522	0.000	0.692	0.000	0.947	0.000	0.000	NaN	0.211	test-label3
GradientBoosting	0.669	0.728	0.6981-0.7589	0.749	0.558	0.702	0.616	0.702	0.749	0.724	0.566	train-label0
GradientBoosting	0.610	0.735	0.7006-0.7691	0.751	0.565	0.354	0.877	0.354	0.751	0.482	0.251	train-label1
GradientBoosting	0.765	0.758	0.7159-0.8005	0.528	0.803	0.299	0.914	0.299	0.528	0.382	0.150	train-label2
GradientBoosting	0.737	0.747	0.6721-0.8218	0.619	0.742	0.091	0.979	0.091	0.619	0.159	0.043	train-label3
GradientBoosting	0.556	0.547	0.3892-0.7047	0.355	0.826	0.733	0.487	0.733	0.355	0.478	0.575	test-label0
GradientBoosting	0.278	0.455	0.2795-0.6305	0.923	0.073	0.240	0.750	0.240	0.923	0.381	0.171	test-label1
GradientBoosting	0.463	0.707	0.5184-0.8947	0.875	0.391	0.200	0.947	0.200	0.875	0.326	0.130	test-label2
GradientBoosting	0.148	0.322	0.0000-0.7020	0.500	0.135	0.022	0.875	0.022	0.500	0.042	0.032	test-label3
MLP	0.519	0.575	0.5406-0.61	0.325	0.788	0.680	0.457	0.680	0.325	0.440	0.595	train-label0

			03									
MLP	0.596	0.583	0.5434-0.6232	0.545	0.613	0.309	0.809	0.309	0.545	0.395	0.251	train-label1
MLP	0.616	0.622	0.5727-0.6712	0.576	0.623	0.196	0.902	0.196	0.576	0.292	0.145	train-label2
MLP	0.703	0.674	0.5922-0.7565	0.548	0.710	0.073	0.974	0.073	0.548	0.129	0.053	train-label3
MLP	0.611	0.631	0.4777-0.7846	0.548	0.696	0.708	0.533	0.708	0.548	0.618	0.573	test-label0
MLP	0.481	0.531	0.3550-0.7069	0.692	0.415	0.273	0.810	0.273	0.692	0.391	0.236	test-label1
MLP	0.741	0.698	0.5061-0.8907	0.500	0.783	0.286	0.900	0.286	0.500	0.364	0.158	test-label2
MLP	0.537	0.548	0.4115-0.6846	0.500	0.538	0.040	0.966	0.040	0.500	0.074	0.048	test-label3

Supplementary Table S10: Detailed predictive efficacy of each model based on traditional machine learning methods.
