# Supplementary material for: Machine Learning and Deep Learning Hybrid Approach Based on Muscle Imaging Features for Diagnosis of Esophageal Cancer
Source: Diagnostics (Basel). 2025 Jul 8;15(14):1730. doi: 10.3390/diagnostics15141730 (PMC12293794; doi:10.3390/diagnostics15141730)
Supplement: Supplementary file 1 [file diagnostics-15-01730-s001.zip › Supplementary Table S11.pdf]

	Model_name	ACC	AUC	95% CI
Esophageal_Pathological Classification				
	densenet121	0.746	0.905	0.8725-0.9371
	densenet121	0.708	0.673	0.5200-0.8255
	densenet201	0.753	0.763	0.7121-0.8145
	densenet201	0.7	0.626	0.4794-0.7733
	resnet152	0.913	0.944	0.9209-0.9668
	resnet152	0.717	0.682	0.5566-0.8081
	resnet50	0.698	0.677	0.6181-0.7351
	resnet50	0.458	0.555	0.4047-0.7063
	resnet18	0.483	0.647	0.5870-0.7072
	resnet18	0.408	0.557	0.4239-0.6908
	vgg13_bn	0.157	0.459	0.3994-0.5190
	vgg13_bn	0.592	0.565	0.4102-0.7190
	vgg19_bn	0.398	0.472	0.4120-0.5327
	vgg19_bn	0.633	0.58	0.4377-0.7228
	ViT	0.658	0.514	0.4501-0.5773
	ViT	0.208	0.587	0.4531-0.7215
	SimpleViT	0.81	0.503	0.4390-0.5667
	SimpleViT	0.242	0.666	0.5545-0.7785
Esophageal plus Stomach_Pathological Classification				
	densenet121	0.939	0.961	0.9362-0.9855
	densenet121	0.89	0.699	0.5519-0.8459
	densenet201	0.855	0.828	0.7785-0.8777
	densenet201	0.856	0.802	0.6716-0.9329

	resnet152	0.77	0.873	0.8296-0.9155
	resnet152	0.864	0.677	0.5157-0.8382
	resnet50	0.765	0.909	0.8758-0.9431
	resnet50	0.737	0.648	0.4928-0.8028
	resnet18	0.765	0.864	0.8233-0.9039
	resnet18	0.703	0.647	0.4679-0.8266
	vgg13_bn	0.167	0.474	0.4128-0.5350
	vgg13_bn	0.305	0.493	0.3654-0.6213
	vgg19_bn	0.78	0.867	0.8250-0.9081
	vgg19_bn	0.788	0.616	0.4658-0.7664
	ViT	0.515	0.5	0.4360-0.5643
	ViT	0.364	0.466	0.3409-0.5902
	SimpleViT	0.591	0.522	0.4588-0.5848
	SimpleViT	0.754	0.581	0.4358-0.7265
Muscle_Pathological Classification				
	densenet121	0.872	0.921	0.8938-0.9486
	densenet121	0.625	0.608	0.4559-0.7609
	densenet201	0.737	0.843	0.8005-0.8863
	densenet201	0.755	0.607	0.4451-0.7683
	resnet152	0.914	0.986	0.9763-0.9961
	resnet152	0.855	0.574	0.4168-0.7307
	resnet50	0.264	0.48	0.4202-0.5408
	resnet50	0.375	0.502	0.3642-0.6394
	resnet18	0.749	0.514	0.4486-0.5790
	resnet18	0.583	0.466	0.3119-0.6203

	vgg13_bn	0.629	0.495	0.4273-0.5626
	vgg13_bn	0.458	0.543	0.4081-0.6773
	vgg19_bn	0.639	0.513	0.4453-0.5811
	vgg19_bn	0.733	0.532	0.3842-0.6794
	ViT	0.627	0.538	0.4790-0.5969
	ViT	0.173	0.5	1.0000-1.0000
	SimpleViT	0.886	0.447	0.3892-0.5054
	SimpleViT	0.175	0.539	0.4211-0.6571
Esophageal_T Staging				
	densenet121	0.516	0.559	0.5165-0.6023
	densenet121	0.73	0.657	0.4943-0.8198
	densenet201	0.588	0.602	0.5597-0.6451
	densenet201	0.419	0.572	0.4085-0.7350
	resnet152	0.591	0.697	0.6594-0.7351
	resnet152	0.514	0.574	0.4350-0.7130
	resnet50	0.425	0.606	0.5654-0.6459
	resnet50	0.703	0.666	0.5137-0.8185
	resnet18	0.731	0.644	0.6012-0.6862
	resnet18	0.473	0.593	0.4337-0.7527
	vgg13_bn	0.56	0.607	0.5641-0.6493
	vgg13_bn	0.459	0.464	0.3086-0.6191
	vgg19_bn	0.554	0.646	0.6049-0.6866
	vgg19_bn	0.73	0.605	0.4215-0.7887
	ViT	0.78	0.522	0.4759-0.5688
	ViT	0.757	0.567	0.4282-0.7063

	SimpleViT	0.605	0.53	0.4848-0.5757
	SimpleViT	0.743	0.551	0.4358-0.6659
Esophageal plus Stomach_T Staging				
	densenet121	0.532	0.532	0.4877-0.5768
	densenet121	0.203	0.349	0.1839-0.5133
	densenet201	0.673	0.838	0.8071-0.8680
	densenet201	0.676	0.705	0.5602-0.8500
	resnet152	0.42	0.569	0.5265-0.6119
	resnet152	0.554	0.667	0.5041-0.8304
	resnet50	0.667	0.507	0.4618-0.5527
	resnet50	0.203	0.204	0.0906-0.3173
	resnet18	0.409	0.522	0.4779-0.5668
	resnet18	0.432	0.443	0.3071-0.5788
	vgg13_bn	0.238	0.478	0.4330-0.5227
	vgg13_bn	0.811	0.686	0.5018-0.8699
	vgg19_bn	0.773	0.472	0.4268-0.5182
	vgg19_bn	0.716	0.686	0.5099-0.8618
	ViT	0.802	0.484	0.4388-0.5300
	ViT	0.446	0.426	0.2794-0.5726
	SimpleViT	0.304	0.497	0.4533-0.5417
	SimpleViT	0.797	0.5	1.0000-1.0000
Muscle_T Staging				
	densenet121	0.592	0.63	0.5881-0.6722
	densenet121	0.5	0.665	0.5280-0.8019

	densenet201	0.236	0.485	0.4407-0.5291
	densenet201	0.473	0.473	0.2935-0.6534
	resnet152	0.706	0.842	0.8118-0.8722
	resnet152	0.784	0.597	0.4305-0.7627
	resnet50	0.447	0.581	0.5384-0.6241
	resnet50	0.541	0.634	0.4807-0.7871
	resnet18	0.663	0.56	0.5154-0.6041
	resnet18	0.351	0.475	0.3094-0.6409
	vgg13_bn	0.47	0.577	0.5359-0.6187
	vgg13_bn	0.365	0.451	0.2980-0.6048
	vgg19_bn	0.545	0.58	0.5384-0.6222
	vgg19_bn	0.811	0.447	0.2634-0.6315
	ViT	0.376	0.483	0.4398-0.5267
	ViT	0.716	0.452	0.2680-0.6360
	SimpleViT	0.433	0.519	0.4748-0.5623
	SimpleViT	0.797	0.516	0.4470-0.5857
Esophageal_N Staging				
	densenet121	0.571	0.576	0.5339-0.6175
	densenet121	0.324	0.489	0.3604-0.6181
	densenet201	0.531	0.565	0.5234-0.6065
	densenet201	0.752	0.442	0.3053-0.5792
	resnet152	0.51	0.555	0.5139-0.5964
	resnet152	0.419	0.498	0.3641-0.6324
	resnet50	0.544	0.538	0.4975-0.5788
	resnet50	0.457	0.474	0.3526-0.5944

	resnet18	0.454	0.493	0.4502-0.5353
	resnet18	0.581	0.613	0.4880-0.7375
	vgg13_bn	0.291	0.484	0.4426-0.5255
	vgg13_bn	0.429	0.492	0.3590-0.6250
	vgg19_bn	0.547	0.571	0.5297-0.6122
	vgg19_bn	0.419	0.473	0.3477-0.5983
	ViT	0.743	0.495	0.4527-0.5364
	ViT	0.762	0.481	0.3749-0.5861
	SimpleViT	0.578	0.493	0.4504-0.5362
	SimpleViT	0.762	0.5	1.0000-1.0000
Esophageal plus Stomach_N Staging				
	densenet121	0.59	0.572	0.5316-0.6134
	densenet121	0.752	0.479	0.3442-0.6128
	densenet201	0.511	0.598	0.5583-0.6385
	densenet201	0.314	0.428	0.2999-0.5566
	resnet152	0.306	0.486	0.4444-0.5268
	resnet152	0.467	0.591	0.4747-0.7068
	resnet50	0.675	0.644	0.6028-0.6856
	resnet50	0.505	0.536	0.4024-0.6686
	resnet18	0.489	0.523	0.4814-0.5655
	resnet18	0.248	0.294	0.1826-0.4049
	vgg13_bn	0.546	0.5	0.4577-0.5417
	vgg13_bn	0.486	0.527	0.4041-0.6499
	vgg19_bn	0.571	0.627	0.5881-0.6659
	vgg19_bn	0.533	0.459	0.3224-0.5951

	ViT	0.715	0.479	0.4365-0.5219
	ViT	0.333	0.42	0.2991-0.5409
	SimpleViT	0.52	0.51	0.4689-0.5519
	SimpleViT	0.714	0.473	0.3362-0.6103
Muscle_N Staging				
	densenet121	0.545	0.525	0.4826-0.5680
	densenet121	0.257	0.43	0.3046-0.5559
	densenet201	0.549	0.609	0.5679-0.6492
	densenet201	0.248	0.388	0.2641-0.5119
	resnet152	0.721	0.75	0.7137-0.7855
	resnet152	0.4	0.402	0.2756-0.5279
	resnet50	0.584	0.583	0.5420-0.6233
	resnet50	0.581	0.517	0.3839-0.6491
	resnet18	0.417	0.604	0.5644-0.6436
	resnet18	0.695	0.456	0.3189-0.5926
	vgg13_bn	0.738	0.45	0.4060-0.4930
	vgg13_bn	0.59	0.598	0.4710-0.7240
	vgg19_bn	0.4	0.563	0.5235-0.6034
	vgg19_bn	0.324	0.38	0.2555-0.5050
	ViT	0.516	0.496	0.4545-0.5381
	ViT	0.695	0.42	0.2830-0.5580
	SimpleViT	0.546	0.522	0.4794-0.5645
	SimpleViT	0.762	0.5	1.0000-1.0000

Supplementary Table S11: Detailed predictive efficacy of each model based on 2d deep learning approach.
