# Supplementary material for: Machine Learning and Deep Learning Hybrid Approach Based on Muscle Imaging Features for Diagnosis of Esophageal Cancer
Source: Diagnostics (Basel). 2025 Jul 8;15(14):1730. doi: 10.3390/diagnostics15141730 (PMC12293794; doi:10.3390/diagnostics15141730)
Supplement: Supplementary file 1 [file diagnostics-15-01730-s001.zip › Supplementary Table S12.pdf]

	Model_name	ACC	AUC	95% CI
Esophageal_Pathological Classification				
	resnet152	0.786	0.49	0.4263-0.5528
	resnet152	0.525	0.589	0.4431-0.7346
	ViT	0.173	0.442	0.3826-0.5023
	ViT	0.417	0.533	0.3780-0.6871
Esophageal plus Stomach_Pathological Classification				
	resnet152	0.561	0.492	0.4254-0.5586
	resnet152	0.763	0.534	0.4269-0.6420
	ViT	0.649	0.477	0.4122-0.5422
	ViT	0.178	0.487	0.3907-0.5832
Muscle_Pathological Classification				
	resnet152	0.786	0.489	0.4270-0.5507
	resnet152	0.825	0.554	0.4672-0.6406
	ViT	0.572	0.655	0.5918-0.7178
	ViT	0.383	0.564	0.4383-0.6893
Esophageal_T Staging				
	resnet152	0.82	0.926	0.9079-0.9445
	resnet152	0.743	0.577	0.4020-0.7528
	ViT	0.746	0.513	0.4658-0.5603
	ViT	0.797	0.396	0.2147-0.5774
Esophageal plus Stomach_T Staging				

	resnet152	0.304	0.491	0.4467-0.5343
	resnet152	0.73	0.482	0.4064-0.5585
	ViT	0.678	0.523	0.4757-0.5700
	ViT	0.595	0.494	0.3346-0.6530
Muscle_T Staging				
	resnet152	0.59	0.571	0.5260-0.6170
	resnet152	0.622	0.641	0.4914-0.7911
	ViT	0.351	0.512	0.4684-0.5563
	ViT	0.743	0.544	0.3584-0.7286
Esophageal_N Staging				
	resnet152	0.51	0.523	0.4815-0.5649
	resnet152	0.752	0.47	0.3356-0.6044
	ViT	0.599	0.511	0.4688-0.5533
	ViT	0.229	0.352	0.2227-0.4818
Esophageal plus Stomach_N Staging				
	resnet152	0.511	0.512	0.4700-0.5533
	resnet152	0.724	0.568	0.4610-0.6750
	ViT	0.667	0.499	0.4557-0.5421
	ViT	0.352	0.358	0.2425-0.4740
Muscle_N Staging				
	resnet152	0.491	0.62	0.5814-0.6581
	resnet152	0.41	0.479	0.3551-0.6024

	ViT	0.677	0.476	0.4337-0.5185
	ViT	0.762	0.507	0.3710-0.6430

Supplementary Table S12: Detailed predicted efficacy of each model based on 3d deep learning approach.
