# Supplementary material for: Machine Learning and Deep Learning Hybrid Approach Based on Muscle Imaging Features for Diagnosis of Esophageal Cancer
Source: Diagnostics (Basel). 2025 Jul 8;15(14):1730. doi: 10.3390/diagnostics15141730 (PMC12293794; doi:10.3390/diagnostics15141730)
Supplement: Supplementary file 1 [file diagnostics-15-01730-s001.zip › Supplementary Table S4.pdf]

	OR	CI	P.value
Age	1.021	0.995-1.047	0.112
Sex	0.517	0.278-0.962	0.037
Height	1.022	0.992-1.052	0.154
Weight	1.036	1.016-1.056	<0.001
BMI	1.107	1.042-1.177	<0.001
Smoking.Status	1.308	0.876-1.954	0.190
Drinking.Status	1.224	0.805-1.861	0.345
T.Staging			
T2	1.776	0.739-4.266	0.199
T3-T4	5.267	2.511-11.050	<0.001
N.Staging			
N1	1.651	0.980-2.781	0.059
N2	4.710	2.955-7.509	<0.001

Supplementary Table S4: Correlation between clinical characteristics and pathological classification of esophageal cancer by univariate logistic regression analysis.
