# Supplementary material for: Machine Learning and Deep Learning Hybrid Approach Based on Muscle Imaging Features for Diagnosis of Esophageal Cancer
Source: Diagnostics (Basel). 2025 Jul 8;15(14):1730. doi: 10.3390/diagnostics15141730 (PMC12293794; doi:10.3390/diagnostics15141730)
Supplement: Supplementary file 1 [file diagnostics-15-01730-s001.zip › Supplementary Table S5.pdf]

| | | OR | CI | P.value |
|---|---|---|---|---|
| Age | | | | |
| | T2 | 1.008 | 0.987-1.030 | 0.462 |
| | T3-T4 | 1.012 | 0.993-1.031 | 0.218 |
| Sex | | | | |
| | T2 | 0.997 | 0.662-1.501 | 0.987 |
| | T3-T4 | 2.119 | 1.444-3.109 | <0.001 |
| Height | | | | |
| | T2 | 1.009 | 0.984-1.034 | 0.477 |
| | T3-T4 | 1.014 | 0.992-1.036 | 0.213 |
| Weight | | | | |
| | T2 | 1.008 | 0.990-1.025 | 0.390 |
| | T3-T4 | 1.000 | 0.985-1.015 | 0.976 |
| BMI | | | | |
| | T2 | 1.017 | 0.961-1.076 | 0.562 |
| | T3-T4 | 0.986 | 0.939-1.035 | 0.570 |
| Smoking.Status | | | | |
| | T2 | 0.689 | 0.473-1.004 | 0.053 |
| | T3-T4 | 0.635 | 0.458-0.880 | 0.006 |
| Drinking.Status | | | | |
| | T2 | 0.899 | 0.609-1.329 | 0.594 |
| | T3-T4 | 0.823 | 0.588-1.151 | 0.256 |
| Pathological Classification | | | | |
| | T2 | 0.563 | 0.234-1.353 | 0.199 |
| | T3-T4 | 0.190 | 0.091-0.398 | <0.001 |
| N.Staging | | | | |
| N0 | T2 | 0.046 | 0.011-0.195 | <0.001 |
| | T3-T4 | 0.124 | 0.028-0.549 | 0.006 |
| N1 | T2 | 0.015 | 0.004-0.060 | <0.001 |
| | T3-T4 | 0.072 | 0.017-0.307 | <0.001 |

**Supplementary Table S5**: Correlation between clinical characteristics and T staging of esophageal cancer by univariate logistic regression analysis.
