# Supplementary material for: Machine Learning and Deep Learning Hybrid Approach Based on Muscle Imaging Features for Diagnosis of Esophageal Cancer
Source: Diagnostics (Basel). 2025 Jul 8;15(14):1730. doi: 10.3390/diagnostics15141730 (PMC12293794; doi:10.3390/diagnostics15141730)
Supplement: Supplementary file 1 [file diagnostics-15-01730-s001.zip › Supplementary Table S6.pdf]

		OR	CI	P.value
Age				
	N1	0.993	0.975-1.010	0.412
	N2-N3	0.983	0.963-1.002	0.084
Sex				
	N1	1.632	1.105-2.411	0.014
	N2-N3	2.328	1.424-3.806	<0.001
Height				
	N1	1.013	0.993-1.035	0.208
	N2-N3	1.039	1.014-1.065	0.002
Weight				
	N1	0.998	0.984-1.013	0.808
	N2-N3	1.002	0.986-1.018	0.798
BMI				
	N1	0.974	0.929-1.021	0.266
	N2-N3	0.958	0.908-1.011	0.119
Smoking.Status				
	N1	0.912	0.675-1.232	0.546
	N2-N3	0.820	0.588-1.144	0.243
Drinking.Status				
	N1	0.763	0.558-1.042	0.089
	N2-N3	0.778	0.548-1.103	0.159
Pathological Classification				
	N1	0.606	0.360-1.020	0.059
	N2-N3	0.212	0.133-0.338	<0.001
T.Staging				
T0-T1	N1	0.203	0.132-0.312	<0.001
	N2-N3	0.015	0.004-0.060	<0.001
T2	N1	0.543	0.383-0.772	<0.001
	N2-N3	0.318	0.208-0.484	<0.001

Supplementary Table S6: Correlation between clinical characteristics and N staging of esophageal cancer by univariate logistic regression analysis.
