# Supplementary material for: Machine Learning and Deep Learning Hybrid Approach Based on Muscle Imaging Features for Diagnosis of Esophageal Cancer
Source: Diagnostics (Basel). 2025 Jul 8;15(14):1730. doi: 10.3390/diagnostics15141730 (PMC12293794; doi:10.3390/diagnostics15141730)
Supplement: Supplementary file 1 [file diagnostics-15-01730-s001.zip › Supplementary Table S7.pdf]

	OR	CI	P.value
Sex	0.793	0.364-1.728	0.559
Weight	1.008	0.962-1.055	0.748
BMI	1.102	0.955-1.270	0.183
T			
T2	1.114	0.450-2.760	0.815
T3-T4	2.827	1.279-6.250	0.010
N.Staging			
N1	1.217	0.702-2.112	0.484
N2	2.894	1.726-4.850	<0.001

Supplementary Table S7: Correlation between clinical characteristics and pathological classification of esophageal cancer by multivariate logistic regression analysis.
