# Supplementary material for: Machine Learning and Deep Learning Hybrid Approach Based on Muscle Imaging Features for Diagnosis of Esophageal Cancer
Source: Diagnostics (Basel). 2025 Jul 8;15(14):1730. doi: 10.3390/diagnostics15141730 (PMC12293794; doi:10.3390/diagnostics15141730)
Supplement: Supplementary file 1 [file diagnostics-15-01730-s001.zip › Supplementary Table S8.pdf]

		OR	CI	P.value
Sex				
	T2	0.731	0.464-1.152	0.177
	T3-T4	1.468	0.947-2.276	0.086
Smoking.Status				
	T2	0.622	0.411-0.942	0.025
	T3-T4	0.717	0.492-1.043	0.082
Pathological Classification				
	T2	0.706	0.289-1.727	0.446
	T3-T4	0.296	0.135-0.647	0.002
N.Staging				
N0	T2	0.046	0.011-0.195	<0.001
	T3-T4	0.017	0.004-0.070	<0.001
N1	T2	0.125	0.028-0.556	0.006
	T3-T4	0.082	0.019-0.349	<0.001

Supplementary Table S8: Correlation between clinical characteristics and T staging of esophageal cancer by multivariate logistic regression analysis.
