# Supplementary material for: Machine Learning and Deep Learning Hybrid Approach Based on Muscle Imaging Features for Diagnosis of Esophageal Cancer
Source: Diagnostics (Basel). 2025 Jul 8;15(14):1730. doi: 10.3390/diagnostics15141730 (PMC12293794; doi:10.3390/diagnostics15141730)
Supplement: Supplementary file 1 [file diagnostics-15-01730-s001.zip › Supplementary Table S9.pdf]

		OR	CI	P.value
Sex				
	N1	1.550	0.913-2.631	0.105
	N2-N3	1.412	0.730-2.731	0.305
Height				
	N1	0.996	0.968-1.024	0.770
	N2-N3	1.029	0.996-1.063	0.089
Pathological Classification				
	N1	0.904	0.523-1.560	0.716
	N2-N3	0.384	0.231-0.639	<0.001
T.Staging				
T0-T1	N1	0.193	0.118-0.316	<0.001
	N2-N3	0.010	0.001-0.074	<0.001
T2	N1	0.553	0.385-0.795	<0.001
	N2-N3	0.347	0.224-0.539	<0.001

Supplementary Table S9: Correlation between clinical characteristics and N staging of esophageal cancer by multivariate logistic regression analysis.
